# Level and determinants of birth preparedness and complication readiness among pregnant women: A cross sectional study in a rural area in Bangladesh

**DOI:** 10.1371/journal.pone.0209076

**Published:** 2018-12-17

**Authors:** Jesmin Pervin, U. Tin Nu, A. M. Q. Rahman, Mahabubur Rahman, Borhan Uddin, Abdur Razzaque, Sandy Johnson, Randall Kuhn, Anisur Rahman

**Affiliations:** 1 International Centre for Diarrhoeal Disease Research, Bangladesh (icddr,b); Mohakhali, Dhaka, Bangladesh; 2 Upazilla Health and Family Planning, Ministry of Health and Family Welfare, Chandpur, Bangladesh; 3 University of Denver, Josef Korbel School of International Studies, Denver, United States of America; 4 University of California–Los Angeles, Jonathan and Karin Fielding School of Public Health, Department of Community Health Sciences, California, United States of America; World Health Organization, SWITZERLAND

## Abstract

**Background:**

Increasing the level of birth preparedness and complication readiness (BP/CR) is one of the key interventions to promote optimal utilization of skilled maternal health services. It is therefore essential to determine the women’s ability to recognize the danger signs and the level of BP/CR. This information can be used to design more effective health interventions.

**Objectives:**

This study was conducted to determine the knowledge in recognition of maternal complications, and the level and factors associated with BP/CR in rural Matlab, Bangladesh.

**Methods:**

A community-based cross-sectional survey was conducted from June- October 2015 on a randomly selected 2262 women who delivered live or stillbirth during the year 2014. A pretested and structured questionnaire was used for data collection. Descriptive and analytical statistical methods were used.

**Results:**

The proportion of study participants with “good knowledge”, measured by the ability to recognise three or more danger signs, in pregnancy and delivery were 26% and 23%, respectively. Out of four BP/CR components, about 15% women saved money, 12% women identified facility for delivery, 9.6% women planned to deliver by skilled birth attendant and 5.3% of women arranged transport. About 12% of women were “well prepared”, measured by planning of at least two components, for skilled childbirth and emergency obstetric complications. In the multivariable logistic regression analysis, asset index, antenatal care (ANC) visits and knowledge of danger signs during pregnancy and delivery were associated with BP/CR. The adjusted odds ratio (OR) of “well prepared” was 4.09 (95% confidence interval [CI]: 2.45–6.82) among women with an asset index of five (richest), compared with women in the asset index of one (poorest). The odds of “well prepared” was six times (OR 5.98, 95% CI: 3.85–9.28) higher for women with four or more ANC visits, compared to women with none or one ANC visit. In comparison to women with “poor knowledge” on maternal danger signs during pregnancy and delivery, the odds ratio of “well prepared” among women with good knowledge during pregnancy and in delivery were 1.95 (95% CI: 1.44–2.63) and 1.74 (95% CI: 1.28–2.36), respectively.

**Conclusion:**

The study revealed a low level of maternal knowledge of danger signs and BP/CR among pregnant women. Further, low socioeconomic status, fewer ANC visits and poor knowledge in recognition of dangers signs on maternal health were associated with low BP/CR. More emphasis should be placed on the quality of information offered to the pregnant women during the prenatal contact and women from low socio-economic gradient should be prioritized to optimize the impact of future BP/CR interventions.

## Introduction

Remarkable achievements have been observed worldwide in improvement in maternal and child survival, the Millennium Development Goals (MDGs) 4 and 5, respectively in the last two decades [1–3]. Globally, the maternal mortality ratio (MMR) and under five child mortality rate (<5CMR) have declined from 385/100,000 live births to an estimated 216/100,000 live births and from 91/1,000 live births to an estimated 43/1,000 live births in 1990 to 2015, respectively [2, 3]. In Bangladesh, MMR dropped from 569 per 100,000 live births in 1990 to 176 in 2015 [3]. Under five child mortality rate decreased from 144 per 1,000 live births to 38 during the same period [2]. Although this achievement is commendable, the rates are still high in comparison to middle- and high-income countries [2, 3]. Under the Sustainable Development Goals (SDGs), member countries of the United Nations, including Bangladesh are committed to reducing the MMR to less than 70/100,000 live births and <5CMR to less than 25/1,000 live births in next 15 years [1, 2].

Bangladesh is one of many countries for which low utilization of health facilities creates an obstacle to realizing these goals. In Bangladesh, it is reported that about 31% of women receive four antenatal visits, 37% of women deliver in health facilities, and 34% women receive post-natal care within two days of post-partum period [4]. One possible reason for low utilization may be that woman and their families need information related to danger signs and birth and complication readiness in order to take effective action. Birth preparedness and complication readiness (BP/CR), a key intervention to promote service utilization, includes having a plan to identify a skilled birth attendant and a health facility preferred by the pregnant woman, saving money, arranging transport and identifying a compatible blood donor during pregnancy [5–10]. The ability of mothers and families to recognize the danger signs during the continuum of pregnancy-delivery-postpartum periods enhances the BP/CR level and assists the family in making appropriate decisions for seeking skilled care, if needed. Earlier studies have suggested that BP/CR intervention raises mothers’ knowledge of maternal and neonatal danger signs, increases antenatal care attendance and facility delivery, and decreases neonatal mortality [11–16].

Studies from Southeast Asia and Africa reported a very low level of birth preparedness and knowledge about complications. Studies from Bangladesh [17], Burkina Faso [18], Ethiopia [19–21], Ghana [22], Nigeria [23] and India [24] reported that the knowledge about danger signs for maternal and neonatal health was low, with between 10% and 30% of women able to recognize at least three or more danger signs. Also less than a quarter of women were well prepared for childbirths [7, 8, 20, 25]. While the available literature suggest low level of BP/CR, several determinants such as socio-economic and demographic factors, uptake of antenatal care, and good knowledge of danger signs were found to influence the level of BP/CR [25–28].

To our knowledge, most of the studies in Bangladesh assessed the BP/CR and knowledge level on recognition of maternal danger signs as part of the evaluation of an intervention [29–31]. Information collected on these important health indicators and the determinants of BP/CR may better inform efforts to improve the level of BP/CR in order to facilitate increase utilization of skilled care, and thereby to improve maternal and neonatal health. The present paper analysed a baseline survey conducted as part of an intervention study to determine the levels of BP/CR, knowledge in recognition of maternal complications and factors related with BP/CR in rural Matlab, Bangladesh.

## Materials and methods

### Study site and design

The study was conducted in two sub-districts–Matlab North and Matlab South—in the district Chandpur. The area is a low-lying deltaic plain intersected by the river Gumti and its tributaries and located 80-85km southeast of Dhaka, the capital of Bangladesh. About 90% of inhabitants are Muslims and the remaining are mostly Hindus. Bangladesh has a reasonable health infrastructure for providing primary health care services with three tiers-Upazila Health Complexes (UHC) at the sub-district level, Union Health and Family Welfare Centres (UHFWC) at the union level and Community Clinics (CC) at the village level [32, 33]. Generally, UHFWC’s provide antenatal care, normal delivery, postnatal care, child care, health education and counselling on the components of BP/CR, maternal and newborn danger signs. Similarly CC’s provide services for maternal and child health care, but not all CC’s provide normal delivery as some health care providers are not trained on skilled birth delivery [32]. Also domiciliary community health workers in village level provide health education on maternal and child health [32, 34]. The UHC offers basic emergency obstetric care and serves as a referral center for UHFWCs and CCs. The UHC covers a population of about 250,000–300,000 populations, while UHFWC and CC covers an average of 25,000 and 6000 population, respectively [32, 34]. Although, the accessibility to these health centers is not a major problem, the utilization is still low in-spite of several efforts to improve access including demand-side financing by the government of Bangladesh [35].

This population-based cross-sectional study utilized baseline survey data that was collected for an ongoing cluster randomized study to implement a model of resource-intensive evidence-based Maternal, Neonatal, and Child Health extension (MNCH-Ext) services into the public health system. Before implementation of the MNCH-Ext programme, we created a listing of all women who delivered live births or stillbirths, or had abortions during the calendar year 2014. Women were identified in the study area by household visits from February to April, 2015. A landmark was identified in each village for selecting the first household and then each household was visited by following a clock-wise direction. Out of 7,018 women identified as having been pregnant, 6,741 women delivered live or stillbirths and 277 women had abortions. For this baseline survey we randomly selected 2,483 women from the recently delivered women available from the listing and approached to them for interviews. Our final sample size of 2,262 excluded 218 women who were not available for interviews or had migrated out of the village, and three women who did not give consent for participation in the study.

### Data collection

A structured interview questionnaire was used for data collection from respondents. All interviews were carried out at the respondent’s home in local language (Bangla). Data collection was done from June to October 2015. Female data collectors were recruited and trained thoroughly on the use of the questionnaire for conducting interviews. The questionnaire included questions on the following information.

Birth preparedness and complication readiness (BP/CR): The questionnaire included questions as to whether or not the mother planned for following basic steps of BP/CR for her last pregnancy: i) identified place for childbirth; ii) identified skilled birth attendant; iii) saved money and iv) arranged transport in case of delivery and obstetric emergency. The BP/CR score was computed from these questions. If a pregnant woman planned at least two components out of four, she was considered as being “well prepared” and the rest were considered “not prepared”.

Knowledge on maternal danger signs: Maternal danger signs were grouped into the phases of pregnancy and delivery. Box 1 below presented the danger signs during different phases [36, 37].

**Box 1:** Danger signs during pregnancy and delivery

Pregnancy	Delivery
• Vaginal bleeding • Convulsion • Severe headache • Blurred vision • Swelling of face • Swelling of hands and feet • Severe lower abdomen pain • Hypertension • Less/no foetal movement	• Excessive vaginal bleeding • Convulsion • Foul smelling discharge • High fever • Baby’s malposition • Hand & feet prolapse • Prolonged labour • Retained placenta • Ruptured uterus • Prolapsed cord • Cord around the neck

Assessment of knowledge on maternal danger signs during pregnancy and delivery was done based on nine and eleven signs, respectively. The knowledge score on maternal danger signs was measured by the total number of correct spontaneous answers given to the key danger signs of the questionnaire. In this study, women who reported spontaneously at least three danger signs for each phases of pregnancy and delivery were considered to have “good knowledge”, and those who reported two or fewer danger signs had “poor knowledge”.

Related covariate information: Data on women’s age, education and socioeconomic status were collected. Numbers of antenatal care visit (ANC) were collected during interview and categorized into “0–1”, “2–3” and “≥4” visits. Educational level was assessed by the number of years completed in school. Economic status was assessed by generating scores through principal- components analysis based on household assets of ownership of a number of consumer items (radio, watch, etc.), dwelling characteristics (wall and roof material), and type of drinking water and toilet facilities. The generated scores were thereafter indexed into quintiles, where one represents the poorest and five the richest [38].

### Data analysis

The results were analysed using both descriptive and analytical approaches. Data were expressed as mean, median and percentages with confidence intervals (CI). To identify factors associated with BP/CR, bivariate logistic regression was used. These results were expressed as the odds ratio (OR) with 95% CI. Factors that were found to have a *p*-value of less than 0.05 in the bivariate analysis and also remained significant (p<0.05) in the multivariable analyses were included in the final model. To understand the robustness of the study findings, we performed stratified analysis by dividing the participants into two groups by median duration of recall period assessed from dates of delivery and interview conducted.

### Ethical considerations

All participants received an explanation of the purpose of the study and signed the written informed consent for participation in the study. The Research and Ethical Review Committees of International Centre for Diarrhoeal Disease Research, Bangladesh approved the study.

## Results

### Characteristics of women

The characteristics of women are presented in Table 1. Out of the total women interviewed about 98% had live birth and 1.7% women reported a stillbirth. Among the live births (2223), 33(1.5%) newborns died within 28 days. The mean age of the women who participated in the study was 24.7(±4.9) and about 12% of women were under the age of 20 years. About two-thirds (65.4%) of the women completed 10 years education and about 50% of the women belonged to asset index one and two. Among the respondents, 26.6% attended four or more ANC visits.

**Table 1:** Characteristics of women participated in the survey in Matlab, Bangladesh.

Variables	Frequency (%)
Age in years	
<20	281 (12.4)
20–29	1560 (69)
≥ 30	421 (18.6)
Women education by year of school attendance	
0–5	520 (23)
6–10	1479 (65.4)
> 10 years	263 (11.6)
Asset index	
One (Poorest)	478 (21.1)
Two	652 (28.8)
Three	389 (17.2)
Four	301 (13.3)
Five (Richest)	442 (19.5)
No. of antenatal care visits	
0–1	781(34.5)
2–3	880 (38.9)
≥ 4	601(26.6)
Pregnancy outcome	
Live Birth	2223 (98.3)
Stillbirth	39 (1.7)

### Knowledge on maternal danger signs during pregnancy and delivery

**Fig 1:**
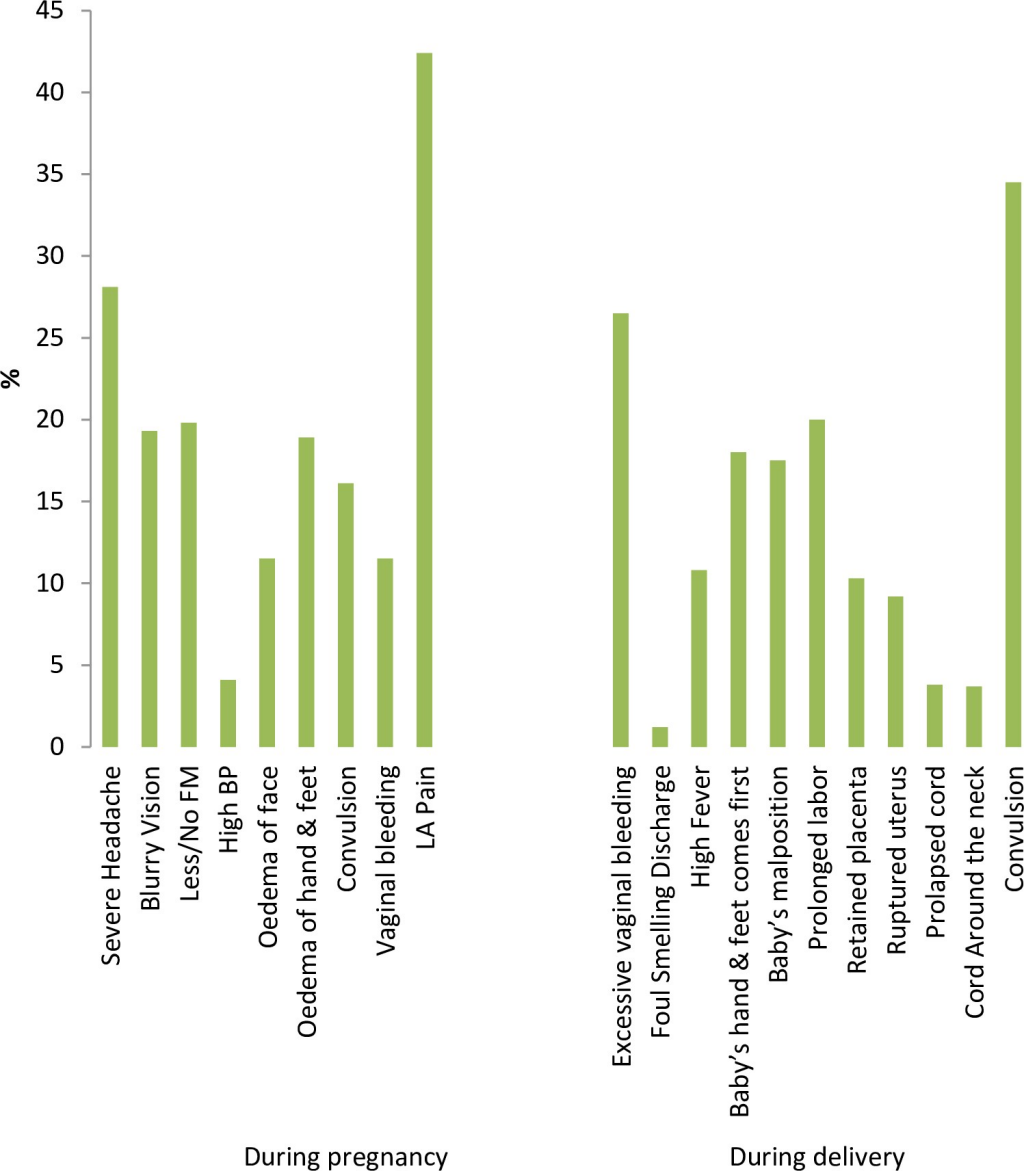
Reported knowledge of study participants on danger signs during pregnancy and delivery in Matlab, Bangladesh (n = 2262). FM: foetal movement; BP: blood pressure; LA: lower abdomen.

The most common spontaneously reported danger signs during pregnancy were pain in lower abdomen (42%), severe headache (28.1%), less/no foetal movement (20%), blurry vision (19.3%), oedema of hand and feet (18.9%). Seven hundred and fifty women (33.2%) could not mention any danger signs while one, two, three and four danger signs during pregnancy were mentioned by 22.9%, 21.1%, 12.1% and 4.7% of the women, respectively (Fig 1).

Out of eleven danger signs of delivery; convulsion (34.5%), excessive per-vaginal bleeding (26.5%), prolonged labour (20%), baby’s hand and feet coming out first (18%) followed by baby’s mal-position (17.5%) were cited by the respondents frequently (Fig 1). Five hundred and fifty women (24.3%) could not express any danger sign while one, two, three and four danger signs of delivery were mentioned by 23.4%, 26.1%, 15.4% and 6.1% of the respondents respectively.

The median scores of knowledge on danger signs during pregnancy and delivery were 2 and 1 respectively. However, 26.1% and 22.8% had “good knowledge” (reported at least three danger signs) during the phases of pregnancy and delivery, respectively. When we changed the cut-off point to 5, we found only 5% and 6% women had good knowledge on pregnancy and delivery, respectively.

### Birth preparedness and complication readiness (BP/CR)

Overall the level of BP/CR was found to be low. Some 76.4% women did not make any plan for birth preparedness. Out of four components, 11.5% women planned only one component while 7.4%, 3.6% and 1.1% of respondents planned for two, three and four components, respectively.

**Fig 2:**
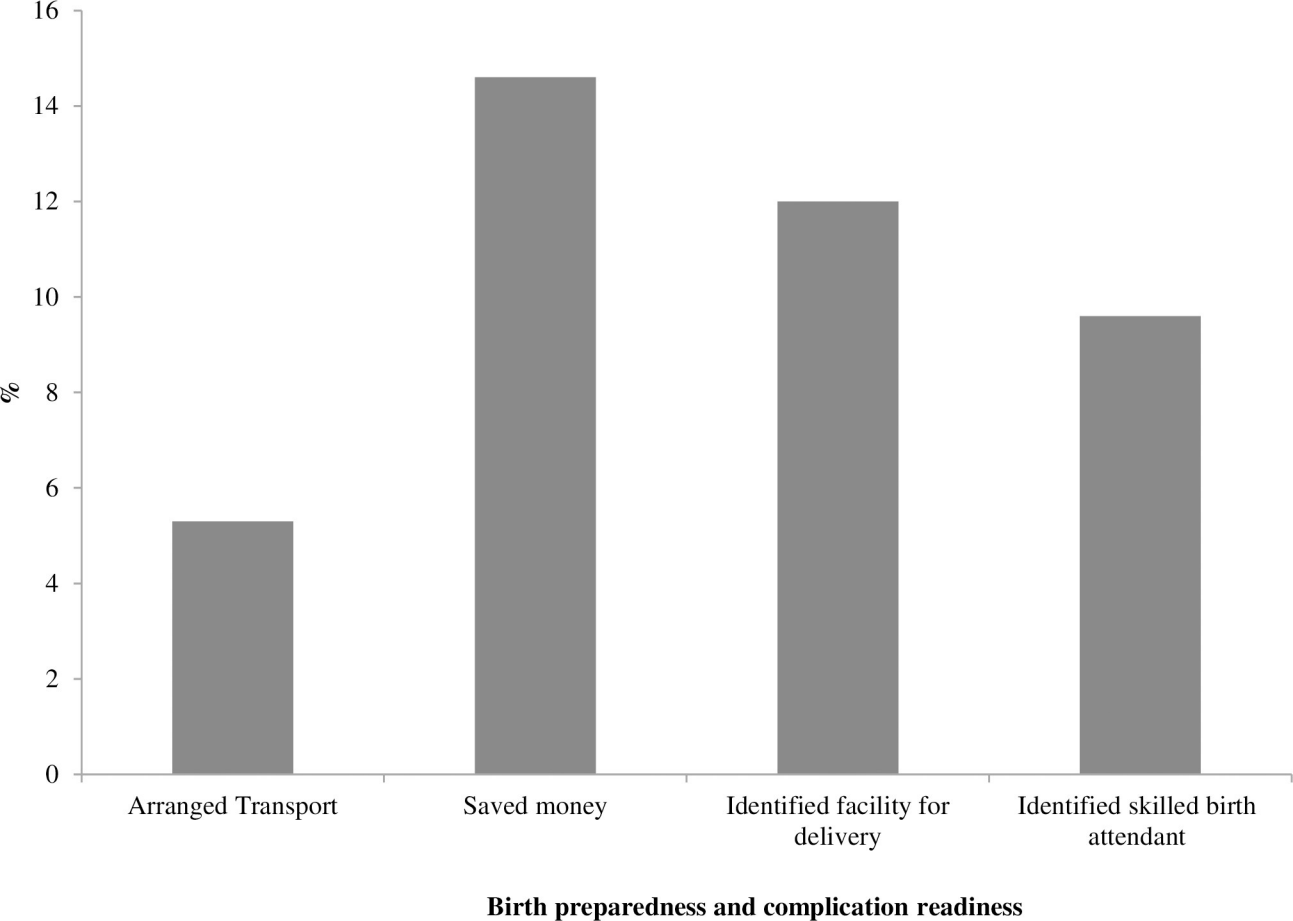
Birth preparedness and complication readiness (BP/CR) among respondents (n = 2262).

A majority of the respondents (88%) reported that they did not make any decisions about where to give birth. The highest level of preparedness was for saving money (about 15%). Only 12% identified a facility for delivery followed by 9.6% pregnant women who planned to be assisted by skilled birth attendant. Preparedness for transportation to reach the health facility was found to be very low (5.3%) during the last pregnancy (Fig 2). As a whole, 12.2% of pregnant women in this study were considered to be well prepared for childbirth based on reporting two or more preparedness activities.

### Factors associated with BP/CR

Out of the factors included in our model, women’s education, asset index, antenatal care visits, and knowledge of danger signs during pregnancy and delivery were found to be significantly associated with BP/CR in the bivariate logistic regression analyses (Table 2). Women’s education was no longer associated with BP/CR when entered into the multivariable model and thus excluded from the final model.

**Table 2:** Association of selected variables with BP/CR.

Variables	BP/CR level		Crude odds ratio (95% confidence interval)	Adjusted odds ratio (95% confidence interval)
	Well prepared n (%)	Not prepared n (%)		
Age in years				
<20	33 (12)	248(12.5)	1.0	
20–29	198 (72)	1362(68.5)	1.09 (.74–1.62)	
≥ 30	44 (16)	377(19)	.88(.54–1.42)	
Women’s education by school attendance in years				
0–5	36 (13.1)	484 (24.4)	1.0	
6–10	179 (65.1)	1300 (65.4)	1.85 (1.27–2.69)	
> 10	60 (21.8)	203 (10.2)	3.97 (2.55–6.20)	
Asset index				
One (Poorest)	21(7.6)	457(23)	1.0	1.0
Two	58(21.1)	594(29.9)	2.12(1.27–3.55)	1.88(1.11–3.20)
Three	49(17.8)	340(17.1)	3.14(1.85–5.33)	2.28(1.32–3.95)
Four	48(17.5)	253(12.7)	4.13(2.42–7.05)	2.84(1.63–4.95)
Five (Richest)	99(36)	343(17.3)	6.28(3.84–10.27)	4.09(2.45–6.82)
No. of antenatal care visits				
0–1	27(9.8)	754(37.9)	1.0	1.0
2–3	93(33.8)	787(39.6)	3.30 (2.13–5.12)	2.45(1.57–3.84)
≥ 4	155 (56.4)	446 (22.4)	9.71(6.34–14.85)	5.98 (3.85–9.28)
Knowledge of danger signs during pregnancy				
Poor Knowledge	142 (51.6)	1529 (77)	1.0	1.0
Good Knowledge	133 (48.4)	458 (23)	3.13 (2.41–4.05)	1.95 (1.44–2.63)
Knowledge of danger signs during delivery				
Poor Knowledge	157 (57.1)	1589 (80)	1.0	1.0
Good Knowledge	118 (42.9)	398 (20)	3.00 (2.31–3.90)	1.74(1.28–2.36)

The adjusted OR of “well prepared” was four (OR 4.09; 95% CI: 2.45–6.82) among women with asset index five (richest), compared with women in asset index one (poorest). To a lesser extent, women in the middle three asset index quintiles also had significantly higher odds of “well prepared”. The odds of “well prepared” was about six times (OR 5.98, 95% CI = 3.85–9.28) higher for women with four or more ANC visits, compared to women with none or one ANC visit. Having 2–3 ANC visits was also found to be favourably associated with well birth prepared. In comparison to women with “poor knowledge” on maternal danger signs during pregnancy and delivery, the odds ratio of “well prepared” among women with “good knowledge” during pregnancy and in delivery were 1.95 (95% CI: 1.44–2.63) and 1.74 (95% CI: 1.28–2.36), respectively (Table 2).

The median duration of recall period was 12 months (range from five to 20 months). We observed a similar increase in BP/CR when we performed stratified analysis by dividing the study women into two groups with median duration of recall period (S1 Table).

## Discussion

Maternal preparedness for birth and complications is an essential precondition for improving perinatal health. The findings of the present study suggest that the study participants were less prepared for childbirth and had a low level of knowledge of danger signs in pregnancy and delivery. ANC visits were associated with a higher level of BP/CR. Economic resources were also essential to BP/CR, with a marked gradient in preparedness across five asset quintiles. After accounting for economic resources, knowledge of danger signs and ANC visits, education was no longer significantly associated with BP/CR.

The strengths of the study include a population-based cross-sectional design, randomly selected study participants that drew from a listing of all recently delivered women in 2014 living within the study area, and a relatively large sample size. The time that had elapsed from delivery time to date of interview ranged from 5–20 months, but our sensitivity analysis showed that recall bias for the relatively longer time did not bias the study findings on BP/CR and knowledge on maternal danger signs (S1 Table). The questionnaire was translated in the local language before conducting interviews, pretesting was performed and data collection was done through well-trained data collectors not involved with clinical care.

There are important limitations to the study. The cross-sectional data do not allow us to describe a causal relationship. In addition, some important determinants like number of previous births and women’s occupation were not collected. We have information on age of the women, which is usually correlated with the birth order. We didn’t find any association between women’s age and BP/CR, and therefore the birth order may not have been associated with BP/CR. As we measured the levels of knowledge at the population level, the lack of birth order information likely would not change the level of BP/CR. Also, those women who experienced pregnancy complications might have better recall compared to women without complications.

The reported knowledge level in this study regarding maternal danger signs was very low but also consistent with other studies [20, 39], although studies in Ethiopia reported a higher level of knowledge in recognition of danger signs [19, 40]. The discrepancies in finding may be due to using of both spontaneous and prompted responses or due to variance in the number of danger signs considered for the assessment of poor or good knowledge [6, 20, 40]. The most common danger signs cited by the respondents were pain in the lower abdomen followed by severe headache during pregnancy as the women might experience these danger signs in their last pregnancy. In the case of delivery, convulsion and excessive vaginal bleeding were mentioned most commonly as danger signs, which might be an indication of awareness by respondents that both bleeding and convulsion are the major causes of maternal mortality in Bangladesh.

This present study also revealed that only 12.2% women were “well prepared” for childbirth and emergency obstetric complications. This finding is in line with the studies done in Ethiopia where ranges were observed between 17–23.3% [7, 25, 41] but also the proportion of well preparedness was much lower than the proportion reported in India, Uganda and Tanzania [6, 27, 42]. However it is difficult to compare our study findings with the other studies due to the differences in the measurement of the BP/CR level and variations in socio cultural environment. In Bangladesh, another study reported that about 24.5% women were observed as well prepared [30]. The difference between this study and our own work could be because the other study was conducted after the implementation of an integrated package of Maternal, Neonatal and Child Survival (MNCS) interventions in the study area.

For childbirth and its complications, the most common observed component of BP/CR in our study was saving money. This finding may be explained by the fact that in case of childbirth and complications, money is needed to get access, facilitate referral to health facility and purchase emergency supplies, which is known by the pregnant women and her family members. Similar finding on birth preparedness was found in other parts of Bangladesh [30], India [42], Tanzania [6], Ethiopia [7] and Uganda [27]. The next most commonly observed BPCR components were that women identified facility (12%) and skilled birth attendant for childbirth (9.6%). This low level of preparedness may be explained by active presence of the traditional birth attendants (TBAs) in rural communities in Bangladesh. The TBAs are considered as trusted health care providers. Also, they can be accessed quickly and are affordable. In addition, cultural and religious beliefs, a lack of female empowerment and the ability for women to make their own decision in terms of healthcare and birth practice could hinder access to a health facility [43, 44].

Our study found that women who attended four or more ANC visits during their last pregnancy were six times as well prepared compared to those who attended 0 or 1 ANC visit. This finding is similar in previous studies [6, 7, 20, 30, 41]. This signifies that antenatal care services inform pregnant women on BP/CR and danger signs that help to motivate the planning of BP/CR components. Significant associations of maternal knowledge during pregnancy and delivery with BP/CR observed in our study were consistent with the findings of studies conducted in Uganda and Ethiopia [8, 27]. This implies that health education on maternal danger signs offered by health care professionals during the prenatal period may contribute to increasing the level of BP/CR. The association of asset index with BP/CR is also in line with other studies [25, 27]. The association may be due to disparities in access to information, the ability to buy health care services, female employment and increase social capital as compared to poorer women.

Bangladesh has experienced significant decline in MMR from 2001 to 2010, but the decline has been stalled since 2010 [45]. The utilization of services has increased since 2010, but this increase has no apparent impact on MMR [45]. To improve the health of women and newborn, World Health Organization (WHO) had already incorporated BP/CR as an integral component of antenatal care by the early 2000s [46]. As there is a wide gap in BP/CR practices, in the new era of the SDG’s to achieve the proposed target, Bangladesh Maternal Health Strategy prioritized BP/CR as an essential intervention of ANC [29]. Community and facility health workers are providing health education on BP/CR, though the efficacy is not consistent perhaps due to outreach and the number of times pregnant women receive this information. Pregnant women and their families need to be sensitized by offering quality information on BP/CR consistently during the prenatal contact at both household and facility levels to maximize the benefits of BP/CR intervention [29, 30]. Further, the respective stakeholders should prioritize outreach to the women from low socio-economic gradient to optimize the impact of the BP/CR intervention.

## Conclusion

Levels of BP/CR and knowledge of obstetric danger signs were found to be surprisingly low in this study area, posing an impediment to further reductions in maternal mortality and morbidity as well as perinatal mortality and morbidity. Further, low socioeconomic status, lower number of ANC attendance and poor knowledge in recognition of dangers signs on maternal health were found as significant predictors for BP/CR. More emphasis should be given on the quality of information offered to the pregnant women and family members during the prenatal contact on BP/CR and knowledge on danger signs. Socioeconomic disparities further suggest the need for improvements in the accessibility and quality of care to low income populations.

## Supporting information

S1 Dataset (SAV)

S1 Questionnaire (PDF)

S1 Table Association of selected variables with BP/CR among the two groups of women by median duration of recall periods. (PDF)
