# Supplementary material for: Level and determinants of birth preparedness and complication readiness among pregnant women: A cross sectional study in a rural area in Bangladesh
Source: PLoS One. 2018 Dec 17;13(12):e0209076. doi: 10.1371/journal.pone.0209076 (PMC6296737; doi:10.1371/journal.pone.0209076)
Supplement: S1 Questionnaire — (PDF) [file pone.0209076.s002.pdf]

Matlab Maternal Neonatal and Child Health Extension Program (MNCH-Ext.)
Form: Baseline Survey: Knowledge, Practice and Coverage

Date: |___|___|| |___|___|| |___|___| Interviewers Code: |___|___| Listing ID# |___|___|___|___|___|___|___|

Interviewer's visit and status

**Visit**	**1**	**2**	**3**	**FINAL VISIT**
DATE	....	....	....	DAY: |___|___| MONTH: |___|___| YEAR: |___|___|___|___|
RESULT				RESULT CODE* |___|___|
				INTERVIEWER'S CODE |___|___|___|
NEXT DATE				TOTAL NUMBER OF VISIT |___|
VISIT TIME				

* RESULT CODES	
1=Interview Complete	5=Woman who has recently delivered is absent
2=No HH member OR competent respondent were present at home at time of HH visit	6=No recently delivered woman found
3=Interview cancelled	7=Others (specify) ____________________
4=Refused to give interview	

General information of respondents woman (delivered live or still birth 1st January 2014 to 31th December 2014)

Name of Woman:				Age: |___|___| Years		
Area:	1=HDSS |___|	2=Non HDSS |___|	Upazila:	1=Matlab North |___|	2=Matlab South |___|	
Block:	1=E	2=F	3=G	4=N/A		|___|
Husband's Name:				Age: |___|___| Years		
Cell Phone # (1) :			Cell Phone # (2):			
Bari Numbe |___|___|		Household Number: |___|___|___|___|___|		Cluster Number: |___|___|		
Address:	Village: ____________	P.O: ____________	Union: ____________			
	UpZ / P.S: ____________	District: ____________				
Asset Score: |___|___|			LMP: |___|___| |___|___| |___|___| (DD MM YY)			
Delivery Date: |___|___| |___|___| |___|___| (DD MM YY)						
Pregnancy Outcome:			1= Alive	2=Stillbirth		|___|
Education Woman |___|___| yrs	Type of education:		1=Secular	2=Moktab	3=NGO 4=Other	
Education Husband |___|___| yrs	Type of education:		1=Secular	2=Moktab	3=NGO 4=Other	

Time interview started: |___|___|:|___|___|

No.	QUESTIONS & FILTERS	CODING RESPONSES	SKIP
**SECTION 1: MOST RECENT BIRTH INFORMATION**			
101	CHECK FACE PAGE: BABY BORN DEAD	1=YES ☐ → 201 2=NO ☐ ↓	|___|
102	From the survey, I know that you recently had a baby. What name was given to that/ those child(ren)?	|________| CHILD 1<br>|________| CHILD 2<br>|________| CHILD 3	|___|

103	What is the status of child now?	**CHILD 1**	**CHILD 2**	**CHILD 3**		|___|
		1=Alive	1=Alive	1=Alive		
		2=Dead	2=Dead	2=Dead		

SECTION 2: PREGNANCY

Now I would like to talk to you about pregnancy in general.

Knowledge:

201	Please tell me what complications may occur during pregnancy that needs medical		**UNPROMPTED**	**PROMPTED**	
		1=Severe Headache	A	M	|___|
		2=Blurry Vision	B	N	|___|
		3=Reduced or Absent Fetal Movement	C	O	|___|
		4=High Blood Pressure	D	P	|___|
	Anything else?	5=Edema of the face	E	Q	|___|
	RECORD ALL RESPONSES	6=Eedema of the hands & feet	F	R	|___|
		7=Convulsions	G	S	|___|
		8=Vaginal Bleeding	H	T	|___|
		9=Lower Abdominal Pain	I	U	|___|
		10=None of Above	Z	Z	|___|

Practices

202	Now I would like to talk you about your <u>last</u> pregnancy. Did you receive check-up/antenatal care during this pregnancy?	1=Yes	|___|
		2=No →	207
		9=Don't Remember →	207
203	How many times do you received ANC?	Times |___|___|	|___|
		99=Don't Know / Don't Remember	

204	Mention chronologically in how many months and from where, and who provided the ANC:	1st ANC=|__|__| months	**WHERE**		**WHO**	
		2nd ANC=|__|__| months	1=Home		1= Doctors	
		3rd ANC=|__|__| months	2=H & FWC		2= Nurse/Midwifes	
		4th ANC=|__|__| months	3=Community Clinic		3= Family Welfare Assistant	
			4=UHC South		4= Family Welfare Visitor	
	RECORD ALL RESPONSES		5=UHC North		5= Traditional Birth Attendant	
			6=Private Clinic Matlab		6= Community Health Care Provider	
			7=MCWC Chandpur		7= Health Assistant	
			8=Private Clinic Chandpur		8= Others (Specify) ____________	
			9=Chandpur District Hospital			
			10=Other District Hospital			
			11=Other MCWC			
			12=Other Private Clinic			
			13=Other UHC			
			14=ICMH, Matuail, Dhaka			
			15=NGO			
			16=Others (Specify)			

205	Now I would like to ask you about your antenatal visits.		**UNPROMPTED**	**PROMPTED**	
	When you were pregnant with (NAME) (last pregnancy), what messages did you receive during at least one of your antenatal check-ups?	1=Breastfeed	A	M	|___|
		2=Danger Signs Maternal	B	N	|___|
		3=Birth Planning	C	O	|___|
		4=Facility Delivery	D	P	|___|
	RECORD ALL RESPONSES	5=Danger Signs Newborn	E	Q	|___|
	THEN PROBE: Did you receive [ANY	6=Basic Care New Born	F	R	|___|

	ADDITIONAL MESSAGES]?	7=Use Cleaning Delivery Kit	G	S	|___|
		8=Other (Specify)	X	X	|___|
206	During pregnancy and before the delivery, did you or your family make any plans for the birth?	1=Yes			|___|
		2=No		→	301
		9=Don't Remember		→	301
207	Which plans did you or your family makes for the birth of (NAME)?		UNPROMPTED	PROMPTED	
	RECORD ALL RESPONSES THEN PROBE: Did you make [ANY REMAINING PLANS]?	1=Identify Transport	A	M	|___|
		2=Save Money	B	N	|___|
		3=Identify Facility For Delivery	C	O	|___|
		4=Identify Provider For Delivery	D	P	|___|
		5=Other (Specify)	X	X	|___|
208	CHECK 207: IF "IDENTIFIED FACILITY FOR DELIVERY ("3") 1=YES ☐ ↓		2=NO ☐	→	210 |___|
209	Where did you plan to give birth to (NAME)?	1=Home			|___|
		2=H & FWC			
		3=Community Clinic			
		4=UHC South			
		5=UHC North			
		6=Private Clinic Matlab			
		7=MCWC Chandpur			
		8=Private Clinic Chandpur			
		9=Chandpur District Hospital			
		10=Other District Hospital			
		11=Other MCWC			
		12=Other Private Clinic			
		13=Other UHC			
		14=ICMH, Matuail, Dhaka			
		15=NGO			
		16=Others (Specify)			
210	CHECK 207: IF "IDENTIFIED PROVIDER FOR DELIVERY" ("4") 1=YES ☐ ↓		2=NO ☐	→	301 |___|
211	Who did you select as the birth attendant?	1= Doctors			|___|
		2= Nurse/Midwifes			
		3= Family Welfare Assistant			
		4= Family Welfare Visitor			
		5= Traditional Birth Attendant			
		6= Community Health Care Provider			
		7= Health Assistant			
		8= Others (Specify)			

No.	QUESTIONS & FILTERS	CODING RESPONSES			SKIP
**SECTION 3: DELIVERY**					
Now I would like to talk to you about childbirth.					
**Knowledge**					
301	Now, please tell me what are the complications in women during child birth that need medical treatment?		UNPROMPTED	PROMPTED	
		1=Excessive Vaginal Bleeding	A	M	|___|
		2=Foul- Smelling Discharge	B	N	|___|

	Anything else?<br><br>RECORD ALL RESPONSES	3=High Fever	C	O	|___|
		4=Baby's Hand or Feet Come First	D	P	|___|
		5=Baby Bad Position/Mal Presentation	E	Q	|___|
		6=Prolong Labour (>12 Hours)	F	R	|___|
		7=Retained Placenta	G	S	|___|
		8=Torn Uterus	H	T	|___|
		9=Proloapsed Cord	I	U	|___|
		10=Cord Around The Neck	J	V	|___|
		11=Convulsions	K	W	|___|
		12=Perineal Tear	L	Y	|___|
		13=None of Above	Z	Z	|___|

Practices

Now I would like to ask you some questions about the delivery of (NAME).

302	Where did you give birth to (NAME) ?	1=Home<br>2=H & FWC<br>3=Community Clinic<br>4=UHC South<br>5=UHC North<br>6=Private Clinic Matlab<br>7=MCWC Chandpur<br>8=Private Clinic Chandpur<br>9=Chandpur District Hospital<br>10=Other District Hospital<br>11=Other MCWC<br>12=Other Private Clinic<br>13=Other UHC<br>14=ICMH, Matuail, Dhaka<br>15=NGO<br>16=Others (Specify)	|___|
303	Who conducted the delivery of (NAME) ?	1= Doctors<br>2= Nurse/Midwifes<br>3= Family Welfare Assistant<br>4= Family Welfare Visitor<br>5= Traditional Birth Attendant<br>6= Community Health Care Provider<br>7= Health Assistant<br>8=Village doctor<br>9= Others (Specify) ________________	|___|
304	Did any one else help with the delivery of (NAME)?	1=Yes<br>2=No →	|___| 306

305	If yes, who helped with the delivery, other than the birth attendant?<br>Any one else?<br>RECORD ALL RESPONSES	1=Mother-In-Law/ Shashuri<br>2=Mother/ Ma<br>3=Sister/Boin<br>4=Sister-In-Law/Jal/ Nonod<br>5=Other Member of in-laws home<br>6=Other Member of father's home<br>7=Other Relatives	|___|

		8=Neighbor<br>9=Don't Remember	
306	CHECK 302: DELIVERED AT HOME?	1=YES [ ] 2=NO [ ] →	314
307	Did you have a clean birth/delivery kit?	1=Yes<br>2=No →	|___| 310
308	If yes, have you used the delivery kit?	1=Yes<br>2=No →	|___| 310

			UNPROMPTED	PROMPTED	
309	What did you uses the birth/ delivery kit for?<br><br>RECORED ALL RESPONSES<br><br>THEN PROBE: Did you use [ANT REMAINING ITEMS]?	1=Plastic Sheet For Delivery	A	M	|___|
		2=Soap To Wash Hands	B	N	|___|
		3=Blade to Cut Cord	C	O	|___|
		4=Thread to Tie Cord	D	P	|___|
		5=Cotton	E	Q	|___|
		6=Gauze	F	R	|___|
		7=Other (Specify)	X	X	|___|

310	Were any of the following procedures performed at the time of delivery?	a. Blood Transfusion	1=Yes	2=No	9=Don't know	|___|
		b. Fluid	1=Yes	2=No	9=Don't know	|___|
		c. Injection	1=Yes	2=No	9=Don't know	|___|
		d. Episiotomy	1=Yes	2=No	9=Don't know	|___|
		e. Received tablet Misoprostol	1=Yes	2=No	9=Don't know	|___|

311	CHECK 310: RECEIVED INJECTION ("C")	1=YES [ ] 2=NO [ ] →	314<br>|___|
312	What was the injection for?<br><br>RECORD ALL RESPONSES	1=Speed Up Labour<br>2=Reduce Bleeding<br>3=Stop Convulsions<br>4=Other (Specify)<br>99=Don't Know	|___|
313	Who pushed the injection?	1= Doctors<br>2= Nurse/Midwifes<br>3= Family Welfare Assistant<br>4= Family Welfare Visitor<br>5= Traditional Birth Attendant<br>6= Community Health Care Provider<br>7= Health Assistant<br>8=Village Doctor<br>9=Paramedic<br>10= Others (Specify)	|___|
314	What position would you prefer for delivery?	1=Squatting Position<br>2=Semi-Squatting Position<br>3=Lying On Back<br>4=Lying On Back With Feet In Stirrups<br>5=Others (Specify)	|___|
315	In what position were you in during the delivery of (NAME)?	1=Squatting Position<br>2=Semi-Squatting Position<br>3=Lying On Back<br>4=Lying On Back With Feet In Stirrups	|___|
316	CHECK 302: DELIVERED AT SC/HOSPITAL?	1=YES [ ] 2=NO [ ] →	318

317	If anything, what did you like about the care provided by the staff of the FWC/UHC?<br><br>PROBE: What else?<br><br>RECORD ALL RESPONSES	1=Good Behavior<br>2=Availability Medicine/ Supply<br>3=Do Not Have to Wait<br>4=Treatment Effective/ Cured<br>5=Willing to Answer My Question<br>6=Affordable<br>7=Clean<br>8=Friendly and Communicates Well<br>9=Other (Specify)<br>98=None Mentioned	|___|
318	If anything, what did you NOT like about the care provided by the staff of the FWC/UHC?<br><br>PROBE: What else?<br><br>RECORD ALL RESPONSES	1=Bad Behavior<br>2=Non-Availability Medicine<br>3=Started Treatment Late<br>4=Very Expensive/ Can Not Afford<br>5=Not Willing to Answer Question<br>6=Not Clean<br>7=Not Friendly and Communicating<br>8=Treatment Not Effective/ Not Cured<br>9=Other (Specify)<br>98=None Mentioned	|___|
Now I would like to talk to you about problems you experienced during your last pregnancy.			
319	Did you experience any of the following problems during the pregnancy, delivery, up to 24 hours after delivery, and /or up to 42 days after delivery of (NAME)?<br><br>RECORD ALL RESPONSES<br>A. Excessive bleeding was so much that it wet your clothes and you feared it was life threatening?<br>B. A high fever<br>C. Bad smelling vaginal discharge?<br>D. Convulsions?<br>E. Prolonged labor (>12 hours)?<br>F. Retained placenta/ placenta expelled late?	PROBLEM — TIMING OF PROBLEM<br><br>A. EXCESSIVE BLEEDING<br>YES ....1 →<br>1=PREGNANCY<br>2=DELIVERY<br>3=24 HOURS<br>4=42 DAYS<br>NO............2 ↓	|___| |___| <br>|___|
		B. HIGH FEVER<br>YES ....1 →<br>1=PREGNANCY<br>2=DELIVERY<br>3=24 HOURS<br>4=42 DAYS<br>NO............2 ↓<br><br>C. BAD SMELLING VAGINAL DISCHARGE<br>YES ....1 →<br>1=PREGNANCY<br>2=DELIVERY	|___| |___| <br>|___| <br>|___| |___|

		3=24 HOURS<br>4=42 DAYS<br>NO............2 ↓	|___|
		D. CONVULSION<br>YES ....1 →	|___|
		1=PREGNANCY<br>2=DELIVERY<br>3=24 HOURS<br>4=42 DAYS	|___|
		NO............2 ↓	|___|
		E. PROLONGED LABOR (> 12 HOURS)<br>YES ....1<br>NO............2 ↓	|___|
		F. RETAINED PLACENTA<br>YES ....1<br>NO............2	|___|
320	CHECK 319: AT LEAST ONE COMPLICATION?	1=YES ☐ ↓ 2=NO ☐ →	332
321	Did you seek any care or treatment for any of these/this complication(s)?<br>PROBE: Any care/ treatment received at home?	1=Yes<br>2=No →	|___| 332
322	Where did you <u>first</u> seek care/treatment for these/this complication(s)?<br><br>RECORD ONLY ONE ANSWER- WHERE WOMAN OR FAMILY <u>FIRST</u> SOUGHT CARE/TREATMENT	1=Home<br>2=H & FWC<br>3=Community Clinic<br>4=UHC South<br>5=UHC North<br>6=Private Clinic Matlab<br>7=MCWC Chandpur<br>8=Private Clinic Chandpur<br>9=Chandpur District Hospital<br>10=Other District Hospital<br>11=Other MCWC	|___|
		12=Other Private Clinic<br>13=Other UHC<br>14=ICMH, Matuail, Dhaka<br>15=NGO<br>16=Paramedic's Home<br>17=Village Doctor's Home<br>18=Pharmacy<br>19=Spitual/Healer Home<br>20=Kobiraj/Hakim Home<br>21=Homeopathic Chember<br>22=Others (Specify)	
323	Who provided this care/treatment?	1= Doctors<br>2= Nurse/Midwifes	|___|

	RECORD ONLY ONE ANSWER- WITH WHOM WOMAN OR FAMILY FIRST SOUGHT CARE/TREATMENT	3= Family Welfare Assistant<br>4= Family Welfare Visitor<br>5= Traditional Birth Attendant<br>6= Community Health Care Provider<br>7= Health Assistant<br>8=Village doctor<br>9=Spiritual healer<br>10=Homeopathic<br>11=Kabiraj/Hakim<br>12=NGO worker<br>13= Others (Specify)	
324	What type of care/treatment did you receive?<br><br>RECORD ALL RESPONSES	1=Medicine From Pharmacy<br>2=Referral From CHRW<br>3=Advice From CHRW<br>4=Spiritual Water/ Substance<br>5=Amulets<br>6=Homeopathic Medicine<br>7=Injection Saline<br>8=Medicine From Doctor/Nurse<br>9=Medicine From Kabiraj/Hakim<br>10=Injection to Speed Labor<br>11=Others (Specify)	|___|
325	Were you referred to any other place for care /treatment of this/these complication(s)?<br>PROBE- CARE INSIDE/OUTSIDE THE HOME?	1=Yes<br>2=No →	|___| 401
326	Where were you referred?	1=H & FWC<br>2=Community Clinic<br>3=UHC South<br>4=UHC North<br>5=Private Clinic Matlab<br>6=MCWC Chandpur<br>7=Private Clinic Chandpur<br>8=Chandpur District Hospital<br>9=Other District Hospital<br>10=Other MCWC	
		11=Other Private Clinic<br>12=Other UHC<br>13=ICMH, Matuail, Dhaka<br>14=NGO<br>15=Others (Specify)	
327	Why were you told to seek treatment at this other place?<br><br>RECORD ALL RESPONSES	1=No Surgery Equipment<br>2=High Blood Pressusre<br>3=For Better Treatment<br>4=Doctor Unavailable<br>5=No Arrangement For Blood Transfusion<br>6=Baby's Upward Position<br>7=Some Part of Baby Came Out<br>8=Baby Stool In Womb	|___|

		9=Cervix did not open<br>10=Other (Specify)<br>99=Don't Know	
328	Did you go to this place?	1=Yes<br>2=No<br>3=Didn't go any where →	|___| 331
329	If yes, how long did you wait to seek care for this complication?	|__|__| HH |__|__| MM	
330	If yes, did any body call you after you want to the referred place, to see how you were doing?	1=Yes<br>2=No<br>99=Don't Know	|___|
331	Why did you not go to the referred place?<br><br>RECORD ALL RESPONSES	1=Woman Didn't Think Necessary<br>2=Husband /Family Didn't Think Necessary<br>3=Facility too far<br>4=No Transport<br>5=No Child Care<br>6=Too Expensive<br>7=Services are Poor Quality<br>8=Didn't Know Where To Go<br>9=No Time to Go<br>10=Money Not Easily Available<br>11=Other (Specify)<br>99=Don't Know	|___|
332	CHECK 207: PLANNED FOR TRANSPORT?	1=YES ☐ ↓ 2=NO ☐ →	334<br>|___|
333	Did you use the transport you identified during pregnancy?	1=Yes<br>2=No	|___|
334	CHECK 207: SAVED MONEY?	1=YES ☐ ↓ 2=NO ☐ →	401<br>|___|
335	Did you use the money that you saved during pregnancy?	1=Yes<br>2=No	|___|

SECTION 4: POSTPARTUM CARE FOR MOTHER

Now I am going to ask you about the medical care you received for your self after the delivery of (NAME)

Knowledge

			UNPROMPTED	PROMPTED	
401	Now, please tell me what are the complications in a woman within 42 days after delivery that need medical treatment?	1=Excessive Vaginal Bleeding	A	M	|___|
		2=Foul- Smelling Discharge	B	N	|___|
		3=High Fever	C	O	|___|
	Anything else?	4=Inverted Nipples	D	P	|___|
		5=Tetanus	E	Q	|___|
	PROBE: IF 'FEVER' OR 'PAIN' ASK ABOUT TYPE OF FEVER OR PAIN.	6=Retained Placenta	F	R	|___|
		7=Severe Abdominal Pain	G	S	|___|
	RECORD ALL RESPONSES	8=Convulsions	H	T	|___|
		9=Engorged Breasts	I	U	|___|
		10=None of Above	X	X	|___|
402	CHECK 302: HOME BIRTH?	1=YES ☐ ↓ 2=NO ☐ →			408<br>|___|

**Practices**			
403	Now, I would like to ask you some questions about your health after the time of delivery.<br><br>After (NAME) was born, did you have a medical check-up?	1=Yes<br>2=No → 501	|___|
404	Where did you receive this <u>first</u> check-up?<br><br>IF RESPONDED MORE THAN ONE PLACE, ASK ABOUT THE PLACE WHERE SHE WENT <u>FIRST</u> FOR CHECK-UP.<br><br>CIRCLE ONLY ONE RESPONSE	1=Home<br>2=H & FWC<br>3=Community Clinic<br>4=UHC South<br>5=UHC North<br>6=Private Clinic Matlab<br>7=MCWC Chandpur<br>8=Private Clinic Chandpur<br>9=Chandpur District Hospital<br>10=Other District Hospital<br>11=Other MCWC<br>12=Other Private Clinic<br>13=Other UHC<br>14=ICMH, Matuail, Dhaka<br>15=NGO<br>16=Others (Specify)	|___|
405	Who checked your health at that time?<br><br>IF RESPONDED MORE THAN ONE PLACE, ASK ABOUT THE PERSON WHO <u>FIRST</u> CHECKED HER HEALTH.<br><br>CIRCLE ONLY ONE RESPONSE	1= Doctors<br>2= Nurse/Midwifes<br>3= Family Welfare Assistant<br>4= Family Welfare Visitor<br>5= Traditional Birth Attendant<br>6= Community Health Care Provider<br>7= Health Assistant<br>8=Village doctor<br>9=Spiritual healer<br>10=Homeopathic<br>11=Kabiraj/Hakim	|___|
		12=NGO worker<br>13= Others (Specify)	
406	How many days or weeks after the delivery did you first check-up take place?<br><br>RECORD '00' DAYS IF SAME DAY	Days |___|___| Weeks |___|___| 99=Don't Know	|___|
407	Did you have this check-up because you were sick or was if a routine check-up?	1=Sick<br>2=Routine<br>3=Both<br>9=Don't Remember	
408	CHECK FACE PAGE: BABY BORN DEAD ('2')?	1=YES → 701 2=NO ↓	|___|

SECTION 5: IMMEDIATE NEWBORN CARE

Now I am going to ask you about care of the newborn baby.

Knowledge of newborn care and danger signs

			UNPROMPTED	PROMPTED	
501	What are some serious health problems that can occur during <u>the first 7 days after birth</u> [illegible]	1=Difficulty or Fast Breathing	A	M	|___|

	that could endanger the life of a newborn baby?<br><br>RECORD ALL RESPONSES	2=Poor Sucking or Feeding	B	N	|___|
		3=Yellow Coloration Skin/Eye	C	O	|___|
		4=Pus/Discharge From Umbilical Cord	D	P	|___|
		5=Pus/Discharge From Eyes	E	Q	|___|
		6=Very Small Baby	F	R	|___|
		7=Convulsions	G	S	|___|
		8=Lethargy, Unconsciousness	H	T	|___|
		9=None of the Above	X	X	|___|
502	Could you name a few basic cares that can be provided to baby just after birth?<br><br>RECORD ALL RESPONSES		**UNPROMPTED**	**PROMPTED**	
		1=Dry and Wrapping	A	M	|___|
		2=Establish Breast Feeding	B	N	|___|
		3=Cord Care	C	O	|___|
		4=Eye Care	D	P	|___|
		5=Others (Specify)	E	Q	|___|
		99=Don't Known	X	X	|___|
503	Are there any benefits of breast feeding?	1=Yes			|___|
		2=No		→	505
504	If yes, what are the benefits of breast feeding in your opinion?<br><br>RECORD ALL RESPONSES<br><br>PROBE: FOR BABY, FOR MOTHER, FOR FAMILY?	**FOR BABY**	**UNPROMPTED**	**PROMPTED**	
		1=Breast Milk Contains All Nutrients	A	M	|___|
		2=For Growth of Baby Up To 6 Months	B	N	|___|
		3=Baby Can Digest Easily	C	O	|___|
		4=Safe For Baby	E	P	|___|
		5=Breast Feed Prevents Infectious Disease	E	Q	|___|
		6=Helps Growth of Brain Of Baby	F	R	|___|
		7=Survival of Baby	G	S	|___|
		**MOTHER**	**UNPROMPTED**	**PROMPTED**	
		1=Makes a Bond Between Mom And Baby	A	M	|___|
		2=Can Reduce Risk Of Pregnancy	B	N	|___|
		3=Uterus Becomes Normal Size Quickly	C	O	|___|
		4=Prevents Anemia By Reducing Post- Partum Bleeding	D	P	|___|
		5=Reduce Breast and Uterine Cancer	E	Q	|___|
		6=Readily Available	F	R	|___|
		**FAMILY**	**UNPROMPTED**	**PROMPTED**	
		1=Saves Money Not Buying Extra Milk/Food	A	M	|___|
		2=Reduces Treatment Cost Of Baby	B	N	|___|
		3=Others (Specify)	C	O	|___|
		4=None of The Above	D	P	|___|
**Practices**					
505	What was the first thing put in (NAME)'s mouth after birth?	1=Colostrum<br>2=Plain Water<br>3=Glucose Water/Sugar<br>4=Honey<br>5=Mustard Oil<br>6=Any Milk Other Than Breast Milk<br>7=Other Liquid<br>8=Other (Specify)			|___|
506	Was (NAME) given colostrums?	1=Yes			|___|

		2=No	
507	When did you start breastfeeding (NAME) after birth?	Minutes |__|__| Hours |__|__| Days |__|__| 98=Never Breastfed<br>99=Don't Know	|__|
508	Did you throw out anything (milk) from your breast before giving breast milk to (NAME)?	1=Yes<br>2=No	|__|
509	Did (NAME) cry immediately after birth?	1=Yes →<br>2=No<br>9=Don't Remember	512<br>|__|
510	Did (NAME) need help breathing or crying shortly after birth?	1=Yes<br>2=No<br>9=Don't Remember →	|__| 512
511	What was done to help (NAME) cry or breath at the time of birth?<br>(DO NOT SUGGEST ANSWERS)<br>RECORD ALL RESPONSES	1=Nothing Mentioned<br>2=Dried The Baby<br>3=Rubbed Back<br>4=Rubbed The Feet<br>5=Mouth to Mouth Resuscitation<br>6=Heated the Cord<br>7=Slapped the Baby<br>8=Held the Baby Upside Down<br>9=Other (Specify)	|__|
512	What was used to cut the umbilical cord?	1=Blade (Normal)<br>2=Sterile Bleed Supplied In Kit<br>3=Bambo Slice/Basher Tol<br>4=Scissor/Kachi<br>5=Sterile Surgical Bleed<br>6=Facility Delivery →<br>7=Other (Specify)<br>99=Don't Know/ Remember	|__| 514

513	Was the item used to cut the cord boiled and /or sterilized?	1=Yes<br>2=No<br>9=Don't Remember	|__|
514	What was used to tie the cord?	1=Sterile Threads From Birth Kit<br>2=Cotton Threads<br>3=Nylon Threads<br>4=Thread of fishing net<br>5=Facility Delivery →<br>6=Other (Specify)<br>9=Don't Remember	|__| 516
515	Was the item used to tie the cord boiled?	1=Yes<br>2=No<br>9=Don't Remember	|__|
516	Was anything applied to the cord immediately after cutting and tying?	1=Yes<br>2=No →<br>9=Don't Remember	|__| 518

517	What was applied to the cord just after cutting the cord?<br><br>RECORD ALL RESPONSES	1=Antibiotic (Powder/Ointment)<br>2=Savlon<br>3=Dettol<br>4=Alcohol/Spirit<br>5=Mustard Oil With Garlic<br>6=Chal Chibano<br>7=Tumeric/ Tumeric Juice/Tumeric peel<br>8=Ginger Juice<br>9=Vermilion<br>10=Boric Powder<br>11=Gentian Violet<br>12=Talcum Powder<br>13=Other (Specify)<br>99=Don't Know	___
518	When was (NAME) bathed for the first time? The first, second, third day of life or later?	1= < 24 Hrs Of Birth<br>2=First Day (> 24 HRS)<br>3=Second Day<br>4=Third Day<br>5=Later<br>9=Don't Remember	___
**SECTION 6: NEWBORN CARE: FIRST MONTH & BREAST FEEDING**			
Now, I would like to ask you some question about what happened during the first months of (NAME's) life.			
601	Before the cord fell off, what was applied to it in the days after delivery?<br><br>Anything else?<br><br>RECORD ALL RESPONSES	1=Nothing<br>2=Antibiotics<br>3=Antiseptic (Savlon)<br>4=Dettol<br>5=Alcohol/Spirit<br>5=Mustard Oil With Garlic<br>6=Ground Rice<br>7=Turmeric/ Turmeric Paste/ Turmeric Powder<br>8=Ginger Juice<br>9=Vermilion	___
		10=Boric Power<br>11=Gentian Violet<br>12=Other Private Clinic<br>13=Other (Specify)<br>14=Nothing<br>99=Don't Know	
602	After (NAME) was born, did any medical persons/ health worker check (NAME)'s health?	1=Yes<br>2=No<br>9=Don't Remember	___<br>2=No → 607
603	How many days or weeks after the delivery did (NAME)'s first check take place?<br><br>RECORD '00' DAYS IS SAME DAY	DAYS AFTER DELIVERY ___ ___<br><br>WEEKS AFTER DELIVERY ___ ___<br><br>99=DON'T KNOW/ REMEMBER	___
604	Who checked (NAME)'s health at that time?	1= Doctors<br>2= Nurse/Midwifes	___

		3= Family Welfare Assistant<br>4= Family Welfare Visitor<br>5= Traditional Birth Attendant<br>6= Community Health Care Provider<br>7= Health Assistant<br>8=Village doctor<br>9=Spiritual healer<br>10=Homeopathic<br>11=Kabiraj/Hakim<br>12=NGO worker<br>13= Others (Specify)	
605	Where did the <u>first</u> check-up take place?	1=Home<br>2=H & FWC<br>3=Community Clinic<br>4=UHC South<br>5=UHC North<br>6=Private Clinic Matlab<br>7=MCWC Chandpur<br>8=Private Clinic Chandpur<br>9=Chandpur District Hospital<br>10=Other District Hospital<br>11=Other MCWC<br>12=Other Private Clinic<br>13=Other UHC<br>14=ICMH, Matuail, Dhaka<br>15=NGO<br>16=Others (Specify)	|___|
606	Was this check because (NAME) was sick or was it a routine check-up?	1=Sick<br>2=Routine<br>3=Both<br>9=Don't Remember	|___|

607	During the first month of life did (NAME) have any of the following problems?	Fever	1=Yes	2=No	9=Don't know	|___|
		Difficulty Breathing	1=Yes	2=No	9=Don't know	|___|
		Jaundice	1=Yes	2=No	9=Don't know	|___|
		Diarrhea	1=Yes	2=No	9=Don't know	|___|
		Umbilical Cord Infection	1=Yes	2=No	9=Don't know	|___|
		Convulsions	1=Yes	2=No	9=Don't know	|___|
		Feeding Problem	1=Yes	2=No	9=Don't know	|___|
608	CHECK 607: AT LEAST ONE PROBLEM?		1=YES ↓	2=NO →		617<br>|___|
609	Did you seek any care or treatment for any of this/these problem(s) (including at home or other place)?	1=Yes<br>2=No →				|___| 617
610	Where did (NAME) <u>first</u> seek care/treatment for these/this problem(s)?<br><br>PROBLEMS ONLY FOR FIRST MONTH OF LIF	1=Home<br>2=H & FWC<br>3=Community Clinic<br>4=UHC South				|___|

	RECORD ONLY ONE ANSWER- WHERE WOMAN OR FAMILY FIRST SOUGHT CARE/TREATMENT	5=UHC North<br>6=Private Clinic Matlab<br>7=MCWC Chandpur<br>8=Private Clinic Chandpur<br>9=Chandpur District Hospital<br>10=Other District Hospital<br>11=Other MCWC<br>12=Other Private Clinic<br>13=Other UHC<br>14=ICMH, Matuail, Dhaka<br>15=NGO<br>16=Matlab icddr,b<br>17=Pharmacy<br>18=Homeopathic<br>19=Others (Specify)	
611	Who provided this care/treatment?<br><br>PROBLEMS ONLY FOR FIRST MONTH OF LIF<br><br>RECORD ONLY ONE ANSWER- WITH WHOM OR FAMILY FIRST SOUGHT CARE/TREATMENT	1= Doctors<br>2= Nurse/Midwifes<br>3= Family Welfare Assistant<br>4= Family Welfare Visitor<br>5= Traditional Birth Attendant<br>6= Community Health Care Provider<br>7= Health Assistant<br>8=Village doctor<br>9=Spiritual healer<br>10=Homeopathic<br>11=Kabiraj/Hakim<br>12=NGO worker<br>13= Others (Specify)	|___|
612	What type of treatment did (NAME) receive?<br><br>RECORD ALL RESPONSES	1=Medicine From Pharmacy<br>2=Referral From FWV/CHCP<br>3=Advice From FWV/CHCP<br>4=Spiritual Water/ Substance	|___|
	PROBLEMS ONLY FOR FIRST MONTH OF LIF	5=Amulets<br>6=Homeopathic Medicine<br>7=Injection Saline<br>8=Medicine From Doctor/Nurse<br>9=Medicine From Kabiraj/Hakim<br>10=Others (Specify)	
613	Was (NAME) referred to any other place for care/treatment of these/this problem(s)?<br><br>PROBLEMS ONLY FOR FIRST MONTH OF LIFE	1=Yes<br>2=No →	|___| 617
614	Where was (NAME) referred?<br><br>PROBLEMS ONLY FOR FIRST MONTH OF LIF	1=Home<br>2=H & FWC<br>3=Community Clinic<br>4=UHC South<br>5=UHC North<br>6=Private Clinic Matlab<br>7=MCWC Chandpur	|___|

		8=Private Clinic Chandpur<br>9=Chandpur District Hospital<br>10=Other District Hospital<br>11=Other MCWC<br>12=Other Private Clinic<br>13=Other UHC<br>14=ICMH, Matuail, Dhaka<br>15=NGO<br>16=Matlab icddr,b<br>17=Others (Specify)	
615	Did you go to referred place?<br>PROBLEMS ONLY FOR FIRST MONTH OF LIF	1=Yes → 2=No	617<br>___
616	If no, why did you not go to the referred place'<br>RECORD ALL RESPONSES<br>PROBLEMS ONLY FOR FIRST MONTH OF LIF	1=Mother didn't think it was necessary<br>2=Husband /Family Didn't think it was necessary<br>3= Other necessary works<br>4=Facility Too Far<br>5=No Transport<br>6=No Child Care<br>7=Too Expensive<br>8=Services are Poor Quality<br>9=Didn't Know Where To Go<br>10=No Time To Go<br>11=Money not easily available<br>12=Other (Specify)<br>99=Don't Know	___
Now, I am going to ask you some questions about the type of food given to (NAME).			
617	For how many month did you breast feed (NAME)?	Months ___ ___<br>Days ___ ___<br>96= Never Breastfed<br>98= Still Breastfeeding<br>99= Don't Know	___

618	Have you ever given (NAME) anything solid/ semi-solid to it or any thing to drink beside breast milk?	1=Yes<br>2=No	
619	At what age did you first give food/drink other than breast milk to (NAME)?	Months ___ ___<br>Days ___ ___<br>99= Don't Know	___
620	What else than breast milk have you ever given to (NAME)?<br>RECORD ALL RESPONSES	1=Plain Water<br>2=Sugar Water/Honey/Juice<br>3=Baby Or Infant Formula<br>4=Cow's Or Goat's Milk<br>5=Other Liquids<br>6=Banana/Papaya/Mango<br>7=Green Leafy Vegetables<br>8=Rice, Wheat, Porridge<br>9=Meat/Fish/Eggs<br>10=Dal	___

		11=Tea<br>12=Allopathic Medicine<br>13=ORS<br>14=Kitchuri<br>15=Other (Specify)<br>16=Nothing	
621	Why did you give foods/drinks other than breast milk to (NAME)?<br><br>RECORD ALL RESPSONSES	1=Baby Older Than 6 Months<br>2=Breast Milk Was Not Enough<br>3=Breast Milk Has Gone Bad Due To Alga<br>4=BF Caused Loose Motion<br>5=Dai/Doctor Prohibited<br>6=Breast Milk Is Too Liquid<br>7=Baby Has Grown- Needs Extra Feed<br>8=Don't Know Benefit Of Exclusive Breastfeeding<br>9=Other (Specify)	|___|
622	CHECK 103: BABY ALIVE?	1=YES ↓ 2=NO →	701<br>|___|
623	How many times did (NAME) eat solid, semi-solid or soft foods other than breast milk in the last <u>24 hour</u>?<br><br>IF 7 OR MORE TIMES, RECORD '7'	TIMES |___|___|	

624	At any time yesterday (last 24 hours) was (NAME) given any of the following?	Water	1= Yes	2= No	|___|
		Sugar /Juice	1= Yes	2= No	|___|
		Infant Formula	1= Yes	2= No	|___|
		Cow Milk	1= Yes	2= No	|___|
		Liquids	1= Yes	2= No	|___|
		Banana	1= Yes	2= No	|___|
		Vegetables	1= Yes	2= No	|___|
		Rice, Wheat	1= Yes	2= No	|___|
		Meat/Fish	1= Yes	2= No	|___|
		Dal	1= Yes	2= No	|___|
		Tea	1= Yes	2= No	|___|
		Allopathic Medicin	1= Yes	2= No	|___|
		ORS	1= Yes	2= No	|___|
		Kitchuri (Lentil Ri	1= Yes	2= No	|___|
		Other (Specify)	1= Yes	2= No	|___|

SECTION 7: PERCEPTIONS OF LOCAL FACILITY

701	Do you know of a health facility where woman can go if she has a health problem or give birth to a baby in your area?	1=Yes<br>2=No →	|___| 801
702	IF YES: where is that?<br><br>RECORD ALL PLACES MENTIONED.	1=H & FWC<br>2=Community Clinic<br>3=UHC South<br>4=UHC North<br>5=Private Clinic Matlab<br>6=MCWC Chandpur<br>7=Private Clinic Chandpur<br>8=Chandpur District Hospital	|___|

		9=Other District Hospital<br>10=Other MCWC<br>11=Other Private Clinic<br>12=Other UHC<br>13=ICMH, Matuail, Dhaka<br>14=NGO<br>15=Others (Specify)	
703	IF MORE THAN ONE FACILITY MENTIONED: which of these health facilities is the closest to here?	1=H & FWC<br>2=Community Clinic<br>3=UHC South<br>4=UHC North<br>5=Private Clinic Matlab<br>6=MCWC Chandpur<br>7=Private Clinic Chandpur<br>8=Chandpur District Hospital<br>9=Other District Hospital<br>10=Other MCWC<br>11=Other Private Clinic<br>12=Other UHC<br>13=ICMH, Matuail, Dhaka<br>14=NGO<br>15=Others (Specify)	|___|
704	If anything, what do you like about the care provided by the staff of the hospital/ health facility?<br>PROBE: What else?<br>RECORD ALL RESPONSES	1=Good Behavior<br>2=Availability of Medicine Supply<br>3=Do Not Have To Wait<br>4=Treatment Effective/ Cured<br>5=Willing To Answer My Questions<br>6=Affordable<br>7=Clean<br>8=Friendly And Communicating<br>9=Other (Specify)<br>10=None Mentioned	|___|
705	If anything, what do you NOT like about the care provided by the staff of the hospital/health facility?<br>PROBE: What else?<br>RECORD ALL RESPONSES	1=Bad Behavior<br>2=Non-Availability Medicine<br>3=Started Treatment Late<br>4=Very Expensive/ Can Not Afford<br>5=Not Willing to Answer my Questions<br>6=Not Clean<br>7=Not Friendly and Communicating<br>8=Treatment not effective/ not cured<br>9=Other (Specify)<br>10=None Mentioned	|___|
706	How do you know about the care provided at this sub-centre/hospital?<br>PROBE: What else?<br>RECORD ALL RESPONSES	1=Previous experience of using this facility<br>2=Heard From Family Members<br>3=Heard From Friends<br>4=Other (Specify)<br>99=Don't Know	|___|

SECTION 8: SOCIO-ECOMIC STATUS

CID of Household Head	
RID of Household Head	
CID of respondent if s/he is not Household Head	

Information on Land:

		Decimal	Kani	Gonda	Kora
801	How much land do your household own?				
	Homestead (including ponds & ditches)	|__|__|__|__|	|__|__|__|__|	|__|__|__|__|	|__|__|__|__|
	Agricultural land	|__|__|__|__|	|__|__|__|__|	|__|__|__|__|	|__|__|__|__|
		1 Kani=	1 Gonda=	1 Kora=	1 Decimal=

Source of Income:

802	a. During the last 12 months what were the sources of income of your household?	1. Agriculture (Own land)	1=Yes	2=No	|__|
		2. Agriculture (Share crops)	1=Yes	2=No	|__|
		3. Land/ pond mortage/ lease/ rent (in/out)	1=Yes	2=No	|__|
		4. Day labourer/ Rickshaw/ Van puller	1=Yes	2=No	|__|
		5. Skilled labourer (driver, carpenter,plumber, electrician)	1=Yes	2=No	|__|
		6. Selling/Catching Fish /Fish Farming	1=Yes	2=No	|__|
		7. Cattle/ Chicken/ Duck/ Poultry/Selling Milk	1=Yes	2=No	|__|
		8. Selling Handicrafts	1=Yes	2=No	|__|
	RECORD ALL RESPONSES	9. Tailoring Work	1=Yes	2=No	|__|
		10. Business (Small)	1=Yes	2=No	|__|
		11. Business (MEDIUM)	1=Yes	2=No	|__|
		12. Various Business (BIG)	1=Yes	2=No	|__|
		13. Paid Employment	1=Yes	2=No	|__|
	b. What was the source of income?	14. Pension	1=Yes	2=No	|__|
		15. Remittance (within country)	1=Yes	2=No	|__|
	Sl No. |__|__|	16. Remittance (Outside country)	1=Yes	2=No	|__|
		17. Food for work	1=Yes	2=No	|__|
		18. Old age/ destitute (allowances)/ VGD/ Other allowances (Freedom fighter), Chairman, Member	1=Yes	2=No	|__|
		19. House/ Shop rent	1=Yes	2=No	|__|
		20. Rent of Taxi, Tempo, Auto	1=Yes	2=No	|__|
		21. Boat/ Rickshaw/ van renting out	1=Yes	2=No	|__|
		22. Interest Business	1=Yes	2=No	|__|
		23. Journalist	1=Yes	2=No	|__|
		24. Others (specify)	1=Yes	2=No	|__|

Food security:

803	Some households may not have food for all members three times every day for all times). Has your household been able to have food for all members for three times every day during the last one year?	1=Yes	2=No	|__|

Housing Facility:

804	a. How many rooms does your household have?					|__|__|
	b. Materials used for Construction of the main dwelling	Items	Roof	Wall	Floor	
		Concrete	1	1	1	|__|
		Tin	2	2	2	|__|
	(Write by observing)	Tin and bamboo	3	3	3	|__|
		Tin and wood	4	4	4	|__|
		Tin and others	5	5	5	|__|
		Bamboo and others	6	6	6	|__|

		Kancha (earth)	7	7	7	|___|
		Wood	8	8	8	|___|
		Others	9	9	9	|___|

Commodities/ Assets

805	Does your household own the following commodities or assets?	Fishing net	1=Yes	2=No	|___|
		Fishing boat	1=Yes	2=No	|___|
		Boat	1=Yes	2=No	|___|
		Shop (Owner)	1=Yes	2=No	|___|
		Rickshaw/ Rickshaw van	1=Yes	2=No	|___|
		Auto rickshaw (CNG/ Battery/ Patrol)	1=Yes	2=No	|___|
	RECORD ALL RESPONSES	Power tiller (Cultivation)	1=Yes	2=No	|___|
		Pump (irrigation / water pump)	1=Yes	2=No	|___|
		Thresher machine	1=Yes	2=No	|___|
		Tools used for agriculture	1=Yes	2=No	|___|
		IPS	1=Yes	2=No	|___|
		Computer	1=Yes	2=No	|___|
		Generator	1=Yes	2=No	|___|
		Solar panel	1=Yes	2=No	|___|
		Tubewell (shallow)	1=Yes	2=No	|___|
		Tubewell (deep)	1=Yes	2=No	|___|
		Quilt/ Blanket	1=Yes	2=No	|___|
		Mattress	1=Yes	2=No	|___|
		Chair/ Table	1=Yes	2=No	|___|
		Dining table	1=Yes	2=No	|___|
		Almirah/ Showcase	1=Yes	2=No	|___|
		Sofa set	1=Yes	2=No	|___|
		Television	1=Yes	2=No	|___|
		Radio/ Tape recorder/ CD/ DVD/ VCD	1=Yes	2=No	|___|
		Land Telephone / Mobile phone	1=Yes	2=No	|___|
		Bi-cycle	1=Yes	2=No	|___|
		Motor cycle	1=Yes	2=No	|___|
		Refrigerator	1=Yes	2=No	|___|
		Fan	1=Yes	2=No	|___|
		Sewing Machine	1=Yes	2=No	|___|
		Others (specify)	1=Yes	2=No	|___|

Livestock

806	Does your household own livestocks?	1=Yes	2=No	|___|
807	If yes, please mention type and number?		**Type**	**Number**
		1	Duck and Chicken	|___|___|
		2	Goat and Sheep	|___|___|
		3	Cow and Buffalo	|___|___|

Latrine

808	Do you have any latrine?		1=Yes	2=No	|___|
809	What types of latrines are used by your household?	Septic tank/ modern toilet	1=Yes	2=No	|___|
		Ring/Slab but waste not drained out	1=Yes	2=No	|___|
		Ring/Slab but waste drained out	1=Yes	2=No	|___|
		Concrete latrine but waste drained out	1=Yes	2=No	|___|
	(Write by observing)	Kancha (Earth) latrine (open)	1=Yes	2=No	|___|

		Open/Bush	1=Yes	2=No	|___|
		Others (Specify)			
**Drinking Water (with in 1 year)**					
810	Does your family have the facility of filter or pure water?		1=Yes	2=No	|___|
811	What are the main sources (most commonly used) of drinking water?	Deep Tubewell	1=Yes	2=No	|___|
		Tubewell (Green)	1=Yes	2=No	|___|
		Tubewell (red)	1=Yes	2=No	|___|
		Tubewell not tested yet	1=Yes	2=No	|___|
		Supply water	1=Yes	2=No	|___|
		Rain water	1=Yes	2=No	|___|
		Pond	1=Yes	2=No	|___|
		River	1=Yes	2=No	|___|
		Ditch/ canal	1=Yes	2=No	|___|
		Others (specify)			
**Source of Light**					
812	Main source of power/ electricity? (Record multiple responses)	Kerosine Oil	1=Yes	2=No	|___|
		Electricity	1=Yes	2=No	|___|
		Solar Panel	1=Yes	2=No	|___|
		Generator	1=Yes	2=No	|___|
		Others	1=Yes	2=No	|___|
**Fuel**					
813	Type of fuel mainly used for cooking (Record multiple responses)	Wood/Wood dust/Paddy Husk	1=Yes	2=No	|___|
		Leaves and straw	1=Yes	2=No	|___|
		Kerosine (stove)	1=Yes	2=No	|___|
		Gas (line)	1=Yes	2=No	|___|
		Gas (Cylinder)	1=Yes	2=No	|___|
		Electric (Oven/ rice cooker/ micro-oven/ grill)	1=Yes	2=No	|___|
**NGO/ Committee**					
814	Is any member of your family the member of an NGO or committee ?		1=Yes	2=No	|___|
815	Did any member of your family take any loans ? (Within the last 6 months)		1=Yes	2=No	|___|
816	If yes, do you/they regularly repay the loan?		1=Yes	2=No	|___|
**Bank Account**					
817	Do you/ any one of your household have an account in a bank?		1=Yes	2=No	|___|

End time: |___|___|:|___|___|

Edited By: **Code:** |___|___| **Entry By:** **Code:** |___|___|

Date: |___|___| |___|___| |___|___| **Date:** |___|___| |___|___| |___|___|

icddr,b

Project: Extending a Successful Maternal, Neonatal, and Child Health Program into the Government Health System in Matlab, Bangladesh
Form: Baseline Survey: Knowledge, Practice and Coverage

Date: |__|__||__|__||__|__|	Interviewers Code: |__|__|	Listing ID# |__|__|__|__|__|__|__|		
**Interviewer's visit and status**				
**Visit**	**1**	**2**	**3**	**FINAL VISIT**
DATE	………………….	………………….	………………….	DAY: |__|__| MONTH: |__|__| YEAR: |__|__|__|__|
RESULT				RESULT CODE* |__|__|
				INTERVIEWER'S CODE |__|__|__|
NEXT DATE				TOTAL NUMBER OF
VISIT TIME				VISIT |__|

* RESULT CODES<br>1=Interview Complete<br>2=No HH member OR competent respondent were present at home at time of HH visit<br>3=Interview cancelled<br>4=Refused to give interview	5=Woman who has recently delivered is absent<br>6=Interview incomplete<br>7=Others (specify) ________________

General information of respondents woman (delivered live or still birth 1st January 2014 to 31th December 2014)

1. Name of Woman:				Age: |__|__| Years		
2. Area:	1=HDSS |__|	2=Non HDSS |__|	Upazila:	1=Matlab North |__|	2=Matlab South |__|	
3. Block:	1=E	2=F		3=G	4=N/A	|__|
4. Husband's Name:				Age: |__|__| Years		
5. Cell Phone # (1) :			6. Cell Phone # (2):			
7. Bari Number: |__|__|		8. Household Number: |__|__|__|__|		9. Cluster Number: |__|__|		
10. Address:	Village: ________ P.O: ________ Union: ________<br>UpZ / P.S: ________ District: ________					
11. LMP: |__|__||__|__||__|__| DD MM YY			12. Delivery Date: |__|__||__|__||__|__| DD MM YY			
13. Pregnancy Outcome:			1= Alive	2=Stillbirth		|__|
14. Education Woman: |__|__| yrs		15. Type of education:	1=Secular	2=Moktab	3=NGO 4=Other	|__|
16. Education Husband: |__|__| yrs		17. Type of education:	1=Secular	2=Moktab	3=NGO 4=Other	|__|

Time interview started: \|__\|__\|:\|__\|__\|

No.	QUESTIONS & FILTERS	CODING RESPONSES	SKIP
**SECTION 1: MOST RECENT BIRTH INFORMATION**			
১০১	প্রথম পৃষ্ঠা দেখুনঃ বাচ্চা মৃত জন্মেছিল?	১=হ্যাঁ ☐→ ২০১ ২=না ☐↓	|__|
১০২	Listing তথ্য থেকে আমি জানি যে, আপনি সম্প্রতি বাচ্চা জন্ম দিয়েছেন। বাচ্চার কি নাম দেওয়া হয়েছিল?	|________| বাচ্চা ১<br>|________| বাচ্চা ২<br>|________| বাচ্চা ৩	|__|

১০৩	বাচ্চাটি এখন কি অবস্থায়?	বাচ্চা ১	বাচ্চা ২	বাচ্চা ৩		|___|
		১=জীবিত<br>২=মৃত	১=জীবিত<br>২=মৃত	১=জীবিত<br>২=মৃত		

SECTION 2: PREGNANCY (গর্ভ ধারণ)

এখন আমি আপনার সঙ্গে গর্ভাবস্থা নিয়ে কথা বলতে চাই।

জ্ঞান

			প্রমৃট ছাড়া	প্রমৃট সহ	
২০১	দয়া করে আমাকে বলুন গর্ভাবস্থায় কি কি সমস্যা হতে পারে যার জন্য চিকিৎসা দরকার হয়?	১=তীব্র মাথা ব্যাথা	A	M	|___|
		২=চোখে ঝাপসা দেখা	B	N	|___|
		৩=গর্ভের বাচ্চার নড়াচড়া কম বা না করা	C	O	|___|
	**এ ছাড়া অন্য কিছু?**	৪=উচ্চ রক্ত চাপ	D	P	|___|
		৫=মুখে পানি আসা বা ফোলা	E	Q	|___|
	**সকল উত্তর রেকর্ড করুন।**	৬=হাতে এবং পায়ে পানি আসা	F	R	|___|
		৭=খিচুনী	G	S	|___|
		৮=যোনীপথে রক্তক্ষরন	H	T	|___|
		৯=তলপেটে ব্যথা	I	U	|___|
		১০=উপরের কোনটিই নয়	Z	Z	|___|

অভ্যাস

এখন আমি আপনার সর্বশেষ গর্ভাবস্থা সম্পর্কে কথা বলব।

২০২	আপনি কি সর্বশেষ গর্ভের সময় গর্ভকালীন সেবা (ANC) পেয়েছিলেন:	১=হ্যাঁ<br>২=না → ৯=মনে নাই →	|___| ২০৬<br>২০৬
২০৩	কতবার আপনি গর্ভকালীন সেবা পেয়েছিলেন?	কতবার |__|__| 99=জানি না / মনে নেই	|___|

			WHERE/ কোথায়		WHO/ কে	
২০৪	কত মাসে, কোথায় এবং কে গর্ভকালীন সেবা দিয়েছিল ধারাবাহিক ভাবে উল্লেখ করুন:	1st ANC=|__|__| months	1=Home		1= Doctors	
		2nd ANC=|__|__| months	2=H & FWC		2= Nurse/Midwifes	
		3rd ANC=|__|__| months	3=Community Clinic		3= Family Welfare Assistant	
		4th ANC=|__|__| months	4=UHC South		4= Family Welfare Visitor	
			5=UHC North		5= Traditional Birth Attendant	
			6=Private Clinic Matlab		6= Community Health Care Provider	
			7=MCWC Chandpur		7= Health Assistant	
			8=Private Clinic Chandpur		8= Others (Specify) ____________	
			9=Chandpur District H			
			10=Other District Hospital			
			11=Other MCWC			
			12=Other Private Clinic			
			13=Other UHC			
			14=ICMH, Matuail, Dhaka			
			15=NGO			
			16=Others (Specify) ____________			

			প্রমৃট ছাড়া	প্রমৃট সহ	
২০৫	এখন আমি আপনার গর্ভকালীন সেবা নিয়ে কিছু প্রশ্ন করতে চাই। শেষবার যখন আপনি গর্ভবতী ছিলেন, গর্ভকালীন স্বাস্থ্য পরীক্ষার (চেক-আপ) সময় কি কি তথ্য অন্ততঃ একবার পেয়েছিলেন?	১=বুকের দুধ খাওয়ানো	A	M	|___|
		২=মায়ের বিপদসমূহ	B	N	|___|

	সকল উত্তর লিপিবদ্ধ করুন।<br>প্রোব করুন: আর কোন তথ্য পেয়েছিলেন ?	৩=প্রসব পরিকল্পনা<br>৪=স্বাস্থ্য কেন্দ্রে প্রসব<br>৫=নবজাতকের বিপদসমূহ<br>৬=নবজাতকের যত্ন<br>৭=পরিস্কার প্রসব কিট<br>৮=অন্যান্য (নির্দিষ্ট করুনঃ)	C<br>D<br>E<br>F<br>G<br>X	O<br>P<br>Q<br>R<br>S<br>X	|___| |___| |___| |___| |___| |___|
২০৬	গর্ভকালীন এবং প্রসবের পূর্বে আপনি অথবা আপনার পরিবার প্রসবের কোন পরিকল্পনা করেছিলেন কি?	১=হ্যাঁ<br>২=না<br>৯=মনে নাই		 →<br>→	|___| ৩০১<br>৩০১
২০৭	আপনি অথবা আপনার পরিবার (নাম) প্রসবের জন্য কি কি পরিকল্পনা নিয়েছিলেন?<br>**সকল উত্তর লিপিবদ্ধ করুন।**<br>**প্রোব করুন: আপনি কি আরও কোন পরিকল্পনা নিয়েছিলেন?**	 ১=যানবাহন নির্ধারণ<br>২=অর্থ সঞ্চয়ের কথা বলা<br>৩=ডেলিভারীর জন্য স্থান নির্ধারণ<br>৪=প্রসব সেবাদানকারী নির্ধারণ<br>৫=অন্যান্য (নির্দিষ্ট করুনঃ)	**প্রমৃট ছাড়া**<br>A<br>B<br>C<br>D<br>X	**প্রমৃট সহ**<br>M<br>N<br>O<br>P<br>X	 |___| |___| |___| |___| |___|
২০৮	২০৭ দেখুনঃ যদি প্রসব কেন্দ্র নির্ধারিন করে থাকে (৩)		১=হ্যাঁ ☐ ↓	২=না ☐ →	২১০<br>|___|
২০৯	আপনি আপনার (নাম) কে কোথায় প্রসব করার পরিকল্পনা করেছিলেন:	1=Home<br>2=H & FWC<br>3=Community Clinic<br>4=UHC South<br>5=UHC North<br>6=Private Clinic Matlab<br>7=MCWC Chandpur<br>8=Private Clinic Chandpur<br>9=Chandpur District Hospital<br>10=Other District Hospital<br>11=Other MCWC<br>12=Other Private Clinic<br>13=Other UHC<br>14=ICMH, Matuail, Dhaka<br>15=NGO<br>16=Others (Specify)			|___|
২১০	২০৭ দেখুনঃ যদি প্রসব সেবাদানকারী নির্ধারণের কথা বলা হয় ('৪')		১=হ্যাঁ ☐ ↓	২=না ☐ →	৩০১<br>|___|
২১১	প্রসব করানোর জন্য কাকে নির্বাচিত করেছিলেন ?	1= Doctors<br>2= Nurse/Midwifes			|___|
		3= Family Welfare Assistant<br>4= Family Welfare Visitor<br>5= Traditional Birth Attendant<br>6= Community Health Care Provider<br>7= Health Assistant<br>8= Others (Specify)			

SECTION 3: DELIVERY

এখন আমি বাচ্চা প্রসবের ব্যাপারে কিছু কথা বলতে চাই।					
জ্ঞান					
৩০১	আপনি কি দয়া করে বলবেন বাচ্চা প্রসবের সময় কোন কোন জটিলতার জন্য চিকিৎসা দরকার হয়।<br><br>আর কিছু?<br><br>সকল উত্তর রেকর্ড করুন।		প্রমৃট ছাড়া	প্রমৃট সহ	
		১=অতিমাত্রায় রক্তস্রাব	A	M	|___|
		২=দুর্গন্ধযুক্ত স্রাব	B	N	|___|
		৩=তীব্র জ্বর	C	O	|___|
		৪=বাচ্চার হাত বা পা আগে আসে	D	P	|___|
		৫=বাচ্চার খারাপ অবস্থান	E	Q	|___|
		৬=বিলম্বিত প্রসব (১২ ঘন্টার বেশী)	F	R	|___|
		৭=গর্ভফুল না পড়া বা থেকে যাওয়া	G	S	|___|
		৮=জরায়ু ছিড়ে যাওয়া	H	T	|___|
		৯=নাড়ী আগে বের হয়ে আসা	I	U	|___|
		১০=গলায় নাড়ী পেচিয়ে থাকা	J	V	|___|
		১১=খিচুনী	K	W	|___|
		১২=প্রসবের রাস্তা ছিড়ে যাওয়া	L	Y	|___|
		১৩=উপরের একটাও না	Z	Z	|___|
অভ্যাস					
আমি এখন আপনার (নাম) প্রসবের ব্যাপারে কিছু প্রশ্ন করতে চাই।					
৩০২	আপনি কোথায় প্রসব করিয়েছিলেন?	1=Home<br>2=H & FWC<br>3=Community Clinic<br>4=UHC South<br>5=UHC North<br>6=Private Clinic Matlab<br>7=MCWC Chandpur<br>8=Private Clinic Chandpur<br>9=Chandpur District Hospital<br>10=Other District Hospital<br>11=Other MCWC<br>12=Other Private Clinic<br>13=Other UHC<br>14=ICMH, Matuail, Dhaka<br>15=NGO<br>16=Others (Specify)			|___|
৩০৩	কে বাচ্চা প্রসব করিয়েছিল?	1= Doctors<br>2= Nurse/Midwifes<br>3= Family Welfare Assistant<br>4= Family Welfare Visitor<br>5= Traditional Birth Attendant			|___|
		6= Community Health Care Provider<br>7= Health Assistant<br>8=Village Doctor<br>9= Others (Specify) ________________			
৩০৪	আর কেউ কি প্রসবে সহায়তা করেছিল?	১=হ্যাঁ<br>২=না →			|___| ৩০৬
৩০৫	যদি প্রসবে সহায়তা করে থাকে তাহলে দাই ছাড়া আর কে সাহায্য	১=শ্বাশুড়ী			|___|

	করোছলেন?<br>আর কেউ ?<br>সকল উত্তর রেকর্ড করুন।	২=মা<br>৩=বোন<br>৪=ননদ /জা<br>৫=শ্বশুড় বাড়ীর অন্যান্য সদস্য<br>৬=বাবার বাড়ীর অন্যান্য সদস্য<br>৭=অন্য আত্মীয়স্বজন<br>৮=প্রতিবেশী<br>৯=মনে নেই	
৩০৬	দেখুন ৩০২ঃ বাড়ীতে প্রসব	১=হ্যাঁ ☐↓ ২=না ☐→	৩১৪
৩০৭	আপনি কি পরিস্কার ডেলিভারী কিট পেয়েছিলেন?	১=হ্যাঁ<br>২=না →	৩১০
৩০৮	উত্তর হ্যাঁ হলে, আপনি কি ডেলিভারী কিট ব্যবহার করেছিলেন?	১=হ্যাঁ<br>২=না →	৩১০
৩০৯	ডেলিভারী কিট আপনি কি কাজে ব্যবহার করেছিলেন?<br>সকল উত্তর রেকর্ড করুন।<br>প্রোব করুন: বাকী জিনিস কি ব্যবহার করেছিলেন ?		

	প্রমৃট ছাড়া	প্রমৃট সহ
১=প্রসবের জন্য প্লাষ্টিক শীট	A	M
২=হাত ধোয়ার জন্য সাবান	B	N
৩=নাড়ী কাটার জন্য ব্লেড	C	O
৪=নাড়ী বাধার জন্য সুতা	D	P
৫=তুলা	E	Q
৬= গজ পিচ	F	R
৭=অন্যান্য (নির্দিষ্ট করুনঃ)	X	X

৩১০	নীচের কোন পদ্ধতি কি প্রসবের সময় অনুসরন করা হয়েছিল ?	রক্ত দেয়া	১= হ্যাঁ	২=না	৯=জানি না
		স্যালাইন দেয়া	১= হ্যাঁ	২=না	৯=জানি না
		ইনজেকশন দেয়া	১= হ্যাঁ	২=না	৯=জানি না
		ইপিসিওটমি	১= হ্যাঁ	২=না	৯=জানি না
		ট্যাবলেট মিজোপ্রষ্টল গ্রহন	১= হ্যাঁ	২=না	৯=জানি না

৩১১	দেখুন ৩১০: ইনজেকশন দেয়া?	১=হ্যাঁ ☐↓ ২=না ☐→	৩১৪
৩১২	ইনজেকশন কি জন্য দেয়া হয়েছিল ?<br>সকল উত্তর রেকর্ড করুন।	১=তাড়াতাড়ি প্রসবের জন্য<br>২=রক্তক্ষরন কমানোর জন্য<br>৩=খিচুনি বন্ধ করতে<br>৪=অন্যান্য (নির্দিষ্ট করুন) ____________<br>৯৯=জানি না	
৩১৩	ইনজেকশন কে দিয়েছিল?	1= Doctors<br>2= Nurse/Midwifes<br>3= Family Welfare Assistant<br>4= Family Welfare Visitor	
		5= Traditional Birth Attendant<br>6= Community Health Care Provider<br>7= Health Assistant<br>8= Village Doctor<br>9=Paramedic<br>10= Others (Specify)	
৩১৪	কিভাবে প্রসব করা আপনার পছন্দ?	১=পায়খানা করার মত বসে	

		২=হাটু গেড়ে বসে<br>৩=চিৎ হয়ে শুয়ে<br>৪=দুই পা দুদিকে ছাড়িয়ে ও চিৎ হয়ে<br>৫=অন্যান্য (নির্দিষ্ট করুন) ________________	
৩১৫	আপনি আপনার (নাম) প্রসবের সময় কি ভাবে ছিলেন?	১=পায়খানা করার মত বসে<br>২=হাটু গেড়ে বসে<br>৩=চিৎ হয়ে শুয়ে<br>৪=দুই পা দুদিকে ছাড়িয়ে ও চিৎ হয়ে<br>৫=অন্যান্য (নির্দিষ্ট করুন) ________________	|___|
৩১৬	দেখুন ৩০২: স্বাস্থ্য কেন্দ্রে / হাসপাতালে প্রসব	১=হ্যাঁ ☐↓ ২=না ☐→	৩১৯<br>|___|
৩১৭	যদি হাসপাতাল/ স্বাস্থ্যকেন্দ্রে সেবাদানকারীর কোন সেবা আপনার ভাল লেগে থাকে তাহলে তা কি ছিল?<br><br>**প্রোব করুন: আর কিছু ?**<br><br>**সকল উত্তর রেকর্ড করুন।**	১=ভাল ব্যবহার<br>২=ঔষধ সরবরাহ পাওয়া<br>৩=অপেক্ষা করতে হয়নি সেবার জন্য<br>৪=চিকিৎসা কার্যকরী ছিল<br>৫=আপনার প্রশ্নের উত্তর দেওয়া<br>৬=খরচ আওতার মধ্যে<br>৭=পরিস্কার<br>৮=বন্ধুভাবাপন্ন এবং কথা বার্তায় ভাল<br>৯=অন্যান্য (নির্দিষ্ট করুন) ________________<br>৯৮=কিছুই উল্লেখ করেন নাই	|___|
৩১৮	যদি হাসপাতাল/ স্বাস্থ্যকেন্দ্রে সেবাদানকারীর কোন সেবা আপনার ভাল না লেগে থাকে তাহলে তা কি ছিল?<br><br>**প্রোব করুন: আর কিছু ?**<br><br>**সকল উত্তর রেকর্ড করুন।**	১=খারাপ ব্যবহার<br>২=ঔষধ না পাওয়া<br>৩=দেরিতে চিকিৎসা শুরু করা<br>৪=খরচ অনেক বেশী/সামর্থের বাইরে<br>৫=জিজ্ঞাসা করলে উত্তর না দেয়া<br>৬=অপরিস্কার<br>৭=বন্ধুভাবাপন্ন না<br>৮=চিকিৎসা কার্যকরী ছিল না / ভাল হয়নি<br>৯=অন্যান্য (নির্দিষ্ট করুন) ________________<br>৯৮=কিছুই উল্লেখ করেন নাই	|___|
**এখন আমি আপনার শেষ গর্ভকালীন অভিজ্ঞতার ব্যাপারে কিছু কথা বলতে চাই।**			
৩১৯	আপনি কি আপনার (নাম) পেটে থাকা অবস্থায়, প্রসবের সময়, প্রসবের পর ২৪ ঘন্টার ভিতরে অথবা প্রসবের পর ৪২ দিন পর্যন্ত নীচের কোন সমস্যায় পড়েছিলেন।<br><br>**সকল উত্তর রেকর্ড করুন।**	PROBLEM TIMING OF PROBLEM	
	A. অতিরিক্ত রক্তক্ষরন যা আপনার কাপড় ভিজিয়ে ফেলেছিল এবং আপনি মৃত্যুর ভয় পেয়েছিলেন?<br>B. তীব্র জ্বর<br>C. যোনী পথে দুর্গন্ধ যুক্ত স্রাব<br>D. খিচুনী<br>E. বিলম্বিত প্রসব (১২ ঘন্টার বেশী)	A. অতিরিক্ত রক্তক্ষরন<br>হ্যাঁ ....1 →<br>1=গর্ভকালীন<br>2=প্রসবের সময়<br>3=প্রসবের ২৪ ঘন্টায়<br>4=প্রসবোত্তর ৪২ দিন	|___| |___|

	F. গর্ভফুল না পড়া বা দেরীতে পড়া	না...........2<br><br>B. তীব্র জ্বর<br>হ্যাঁ ....1<br>=গর্ভকালীন<br>2=প্রসবের সময়<br>3=প্রসবের ২৪ ঘন্টায়<br>4=প্রসবোত্তর ৪২ দিন<br>না...........2<br><br>C. দুর্গন্ধযুক্ত স্রাব যোনী পথে<br>হ্যাঁ ....1<br>=গর্ভকালীন<br>2=প্রসবের সময়<br>3=প্রসবের ২৪ ঘন্টায়<br>4=প্রসবোত্তর ৪২ দিন<br>না...........2<br><br>D. খিচুনী<br>হ্যাঁ ....1<br>=গর্ভকালীন<br>2=প্রসবের সময়<br>3=প্রসবের ২৪ ঘন্টায়<br>4=প্রসবোত্তর ৪২ দিন<br>না...........2<br><br>E. বিলম্বিত প্রসব (১২ ঘন্টার বেশী)<br>হ্যাঁ ....1<br>না...........2<br><br>F. গর্ভফুল না পড়া<br>হ্যাঁ ....1<br>না...........2	
৩২০	দেখুন ৩১৯ অন্তত: ১টি জটিলতা ?	১=হ্যাঁ ২=না	৩৩২
৩২১	আপনি এই জটিলতার জন্য কোন সেবা/চিকিৎসা নিয়েছিলেন?<br><br>**প্রোব ঃ বাড়ীতে কোন সেবা/চিকিৎসা নিয়েছিলেন ?**	১=হ্যাঁ<br>২=না	৩৩২

৩২২	আপনি এই জটিলতার সেবা / চিকিৎসার জন্য প্রথম কোথায় গিয়েছিলেন?<br><br>একটি উত্তর রেকর্ড করুন যেখানে মহিলা বা তার পরিবার প্রথম সেবা / চিকিৎসা নিয়েছিলেন।	1=Home<br>2=H & FWC<br>3=Community Clinic<br>4=UHC South<br>5=UHC North<br>6=Private Clinic Matlab<br>7=MCWC Chandpur	

		8=Private Clinic Chandpur<br>9=Chandpur District Hospital<br>10=Other District Hospital<br>11=Other MCWC<br>12=Other Private Clinic<br>13=Other UHC<br>14=ICMH, Matuail, Dhaka<br>15=NGO<br>16=Paramedic's Home<br>17=Village Doctor's Home<br>18=Pharmacy<br>19=Spiritual Healer Home<br>20=Kobiraj/Hakim Home<br>21=Homeopathic Chamber<br>22=Others (Specify)	
৩২৩	এই চিকিৎসা / সেবা কে দিয়াছিল?<br><br>একটি উত্তর রেকর্ড করুন যার নিকট হতে মহিলা / তার পরিবার প্রথম সেবা / চিকিৎসা পেয়েছিল।	1= Doctors<br>2= Nurse/Midwifes<br>3= Family Welfare Assistant<br>4= Family Welfare Visitor<br>5= Traditional Birth Attendant<br>6= Community Health Care Provider<br>7= Health Assistant<br>8=Village Doctor/ Pharmacy<br>9=Spiritual Healer<br>10=Homeopathic<br>11=Kabiraj/Hakim<br>12=NGO worker<br>13= Others (Specify)	|___|
৩২৪	কি ধরনের **সেবা** / চিকিৎসা আপনি পেয়েছিলেন ?<br><br>**সকল উত্তর রেকর্ড করুন।**	১=ফার্মেসী থেকে ঔষধ<br>২=FWV/CHCP থেকে রেফারেল<br>৩=FWV/CHCP থেকে পরামর্শ<br>৪=পানি পড়া / দ্রব্য<br>৫=তাবিজ<br>৬=হোমিও ঔষধ<br>৭=শিরায় স্যালাইন<br>৮=ডাক্তার / নার্স এর নিকট হতে ঔষধ<br>৯=কবিরাজি/ হেকিমি ঔষধ	|___|
		১০=প্রসব তাড়াতাড়ির জন্য ইনজেকশন<br>১১=অন্যান্য (নির্দিষ্ট করুন) ____________________	
৩২৫	এই সমস্যার জন্য আপনাকে কি কোথাও রেফার করা হয়েছিল?<br><br>**প্রোবঃ বাড়ীতে বা বাড়ীর বাইরের সেবা?**	১=হ্যাঁ<br>২=না →	|___| ৪০১
৩২৬	আপনাকে কোথায় রেফার করা হয়েছিল?	1=H & FWC<br>2=Community Clinic	|___|

		3=UHC South<br>4=UHC North<br>5=Private Clinic Matlab<br>6=MCWC Chandpur<br>7=Private Clinic Chandpur<br>8=Chandpur District Hospital<br>9=Other District Hospital<br>10=Other MCWC<br>11=Other Private Clinic<br>12=Other UHC<br>13=ICMH, Matuail, Dhaka<br>14=NGO<br>15=Others (Specify)	
৩২৭	আপনাকে ঐ স্থানে কেন চিকিৎসা নিতে যেতে বলা হয়েছিল?<br><br>**সকল উত্তর রেকর্ড করুন।**	১=সার্জারীর যন্ত্রপাতি নাই<br>২=উচ্চ রক্ত চাপ<br>৩=উন্নত চিকিৎসার জন্য<br>৪=ডাক্তার ছিলেন না তাই<br>৫=রক্ত সঞ্চালনের ব্যবস্থা নাই<br>৬=বাচ্চা পেটের উপর অংশে ছিল<br>৭=বাচ্চার হাত, পা বা অন্য কিছু বেরিয়ে এসেছিল<br>৮=বাচ্চা ভিতরেই পায়খানা করেছিল<br>৯=জড়ায়ুর মুখ খোলে নাই<br>১০=অন্যান্য (নির্দিষ্ট করুন) ____________<br>৯৯=জানি না	___
৩২৮	আপনি কি ঐ স্থানে গিয়েছিলেন ?	১=হ্যাঁ<br>২=না, অন্য কোথাও গিয়েছিলাম<br>৩=কোথাও যায়নি →	___<br><br>৩৩১
৩২৯	যদি হ্যাঁ হয়, তবে কত সময় পর্যন্ত আপনাকে এই সমস্যায় সেবা নিতে অপেক্ষা করতে হয়েছিল?	___ ___ ঘন্টা ___ ___ মিনিট	
৩৩০	যদি হ্যাঁ হয়, আপনি কেমন আছেন জানার জন্য কেউ আপনার খোঁজ নিয়েছিল কি-না?	১=হ্যাঁ<br>২=না<br>৯৯=জানি না	___
৩৩১	রেফারের স্থানে আপনি কেন যাননি ?<br><br>**সকল উত্তর রেকর্ড করুন।**	১=মা প্রয়োজন মনে করেন নি<br>২=স্বামী / পরিবার প্রয়োজন মনে করেন নি<br>৩=হাসপাতাল অনেক দূরে<br>৪=কোন যানবাহন ছিল না<br>৫=বাচ্চা দেখা শুনা করার কেউ ছিল না	___
		৬=খরচ অনেক বেশী<br>৭=সেবা নিম্ন মানের<br>৮=জানতাম না কোথায় যেতে হবে<br>৯=যাবার সময় ছিল না<br>১০=টাকা ছিল না<br>১১=অন্যান্য (নির্দিষ্ট করুন) ____________<br>৯৯=জানি না	

৩৩২	২০৭ দেখুনঃ যানবাহনের পরিকল্পনা করেছিলেন?	১=হ্যাঁ ↓ ২=না →	৩৩৪
৩৩৩	গর্ভকালীন সময় যে যানবাহন ব্যবহারের কথা ভেবেছিলেন সেটি কি ব্যবহার করেছিলেন ?	১=হ্যাঁ<br>২=না	
৩৩৪	২০৭ দেখুনঃ অর্থ সঞ্চয় করে ছিলেন?	১=হ্যাঁ ↓ ২=না →	৪০১
৩৩৫	আপনি কি সঞ্চিত অর্থ প্রসবের সময় ব্যবহার করেছিলেন ?	১=হ্যাঁ<br>২=না	

SECTION 4: POSTPARTUM CARE FOR MOTHER (মায়ের প্রসবোত্তর সেবা)

আমি (নাম) প্রসবের পর আপনার চিকিৎসা গ্রহণ নিয়ে কিছু প্রশ্ন করবো।

জ্ঞান

			প্রমৃট ছাড়া	প্রমৃট সহ	
৪০১	দয়া করে বলবেন কি বাচ্চা প্রসবের ৪২ দিন এর ভিতর কি কি সমস্যা হতে পারে যার জন্য চিকিৎসার নেয়া দরকার হয়?	১=অতিরিক্ত রক্তক্ষরন	A	M	
		২=দুর্গন্ধযুক্ত স্রাব	B	N	
		৩=তীব্র জ্বর	C	O	
	**আর কিছু?**	৪=স্তনের বোটা চামড়ার ভিতর ঢুকে যাওয়া	D	P	
		৫=ধনুষ্টংকার	E	Q	
	প্রোব ঃ যদি জ্বর বা ব্যথার কথা বলে, জিজ্ঞেস করুন কি রকমের জ্বর বা ব্যথা	৬=গর্ভফুল বের না হওয়া	F	R	
		৭=তীব্র পেট ব্যথা	G	S	
	**সকল উত্তর রেকর্ড করুন।**	৮=খিচুনী	H	T	
		৯=স্তন ফুলে যাওয়া	I	U	
		১০=উপরের কোনটাই না	X	X	
৪০২	৩০২ দেখুনঃ বাড়ীতে প্রসব?	১=হ্যাঁ ↓ ২=না →			৪০৮

অভ্যাস

প্রসবের পরের স্বাস্থ্য সম্পর্কে জানতে এখন আপনাকে কিছু প্রশ্ন করব।

৪০৩	আপনার (নাম) জন্মের পর আপনি কোন মেডিকেল চেক-আপ করিয়েছিলেন?	১=হ্যাঁ<br>২=না →	৫০১
৪০৪	**আপনি ১ম চেক-আপ কোথায় করিয়েছিলেন?**<br><br>যদি একের অধিক উত্তর হয় তাহলে মা'কে জিজ্ঞাসা করুন প্রথম কোথায় গিয়েছিলেন।<br><br>**একটি উত্তরে গোল দাগ দিন।**	1=Home<br>2=H & FWC<br>3=Community Clinic<br>4=UHC South<br>5=UHC North<br>6=Private Clinic Matlab<br>7=MCWC Chandpur	
		8=Private Clinic Chandpur<br>9=Chandpur District Hospital<br>10=Other District Hospital<br>11=Other MCWC<br>12=Other Private Clinic<br>13=Other UHC<br>14=ICMH, Matuail, Dhaka	

		15=NGO<br>16=Others (Specify)	
৪০৫	আপনার স্বাস্থ্য তখন কে পরীক্ষা করেছিল?<br><br>যদি উত্তর একাধিক হয় তাহলে জিজ্ঞাসা করুন কে প্রথম পরীক্ষা করেছিল ?<br><br>**একটি মাত্র উত্তরে গোল দাগ দিন।**	1= Doctors<br>2= Nurse/Midwifes<br>3= Family Welfare Assistant<br>4= Family Welfare Visitor<br>5= Traditional Birth Attendant<br>6= Community Health Care Provider<br>7= Health Assistant<br>8=Village doctor/Pharmacy<br>9=Spiritual healer<br>10=Homeopathic<br>11=Kabiraj/Hakim<br>12=NGO worker<br>13= Others (Specify)	|___|
৪০৬	প্রসবের কতদিন বা সপ্তাহ পর আপনি প্রথম চেকআপ করিয়েছিলেন?<br><br>**'00' রেকর্ড করুন যদি প্রসবের দিন চেক আপ করে থাকেন ।**	দিন |___|___| সপ্তাহ |___|___| ৯৯=জানি না/মনে নাই	|___|
৪০৭	আপনি যে চেকআপ করিয়েছিলেন এটা কি অসুস্থতার জন্য নাকি রুটিন চেকআপ ?	১=অসুস্থ<br>২=রুটিন<br>৩=উভয়<br>৯=মনে নেই	|___|
৪০৮	প্রথম পৃষ্ঠা দেখুনঃ বাচ্চা মৃত জন্মেছিল? (২)	১=হ্যাঁ → ৭০১ ২=না ↓	|___|

SECTION 5: IMMEDIATE NEWBORN CARE (নবজাতকের তাৎক্ষনিক পরিচর্যা)

আমি এখন আপনাকে নবজাতকের সেবা সম্পর্কে কিছু প্রশ্ন করবো :

জ্ঞান

			প্রমৃট ছাড়া	প্রমৃট সহ	
৫০১	কি কি গুরুতর স্বাস্থ্য সমস্যা জন্মের ৭ দিনের ভিতর হয়, যা থেকে নবজাতকের মৃত্যুর আশংকা আছে।	১=শ্বাস কষ্ট অথবা তাড়াতাড়ি শ্বাস নেয়া	A	M	|___|
		২=কম দুধ টানা বা খাওয়া	B	N	|___|
		৩=চামড়া বা চোখ হলুদ হওয়া	C	O	|___|
	**সকল উত্তর লিপিবদ্ধ করুন।**	৪=নাভীতে পুঁজ থাকা বা নির্গত হওয়া	D	P	|___|
		৫=চোখে পুঁজ থাকা বা নির্গত হওয়া	E	Q	|___|
		৬=খুবই ছোট বাচ্চা	F	R	|___|
		৭=খিচুনী	G	S	|___|
		৮=কম নড়াচড়া / অজ্ঞান	H	T	|___|
		৯=উপরের কোনটাই না	X	X	|___|
৫০২	আপনি কি কিছু প্রাথমিক সেবার কথা বলতে পারেন যা নবজাতককে জন্মের পর পরই দিতে হয়:		প্রমৃট ছাড়া	প্রমৃট সহ	
		১=শরীর মোছানো এবং কাপড়ে জড়ানো	A	M	|___|
	**সকল উত্তর লিপিবদ্ধ করুন।**	২=বুকের দুধ খাওয়ানো শুরু করা	B	N	|___|
		৩=নাড়ীর যত্ন	C	O	|___|
		৪=চোখের যত্ন	D	P	|___|
		৫=অন্যান্য (নির্দিষ্ট করুন)	E	Q	|___|
		৯৯=জানি না	X	X	|___|

৫০৩	বুকের দুধের কি কোন উপকারিতা আছে?	১=হ্যাঁ<br>২=না	|___| ৫০৫
৫০৪	যদি হ্যাঁ হয়, আপনার মতে বুকের দুধ পান করার উপকারিতা কি কি?<br><br>**সকল উত্তর রেকর্ড করুন।**<br><br>**প্রোব করুন: বাচ্চার জন্য, মায়ের জন্য ও পরিবারের জন্য**	**বাচ্চার জন্য:** **প্রম্‌ট ছাড়া** **প্রম্‌ট সহ** →<br>১=বুকের দুধে পুষ্টি থাকে A M<br>২=৬ মাস পর্যন্ত বাচ্চার বেড়ে ওঠার জন্য সকল পুষ্টি মায়ের দুধে থাকে B N<br>৩=বাচ্চা সহজেই হজম করতে পারে C O<br>৪=বাচ্চার জন্য নিরাপদ E P<br>৫=বুকের দুধ সংক্রামক ব্যধি থেকে শিশুকে রক্ষা করে E Q<br>৬=শিশুর ব্রেইন গঠনে সাহায্য করে F R<br>৭=বাচ্চার জীবন বাচায় G S	|___| |___| |___| |___| |___| |___| |___|
		**মায়ের জন্য:** **প্রম্‌ট ছাড়া** **প্রম্‌ট সহ**<br>১=মা ও শিশুর মধ্যে বন্ধন তৈরী করে A M<br>২=গর্ভধারণ থেকে মাকে বিরত রাখে B N<br>৩=জরায়ু দ্রুত স্বাভাবিক অবস্থায় ফিরে আসে C O<br>৪=প্রসবোত্তর রক্তক্ষরন কমিয়ে রক্ত স্বল্পতাকে প্রতিহত করে D P<br>৫=স্তন এবং জরায়ুর ক্যান্সার কমিয়ে দেয় E Q<br>৬=বানানো লাগেনা বা সব সময় প্রস্তুত থাকে F R	|___| |___| |___| |___| |___| |___|
		**পরিবারের জন্য:** **প্রম্‌ট ছাড়া** **প্রম্‌ট সহ**<br>১=দুধ/খাদ্য কেনার জন্য আলাদা টাকার দরকার হয় না (খরচ কমায়) A M<br>২=শিশুর চিকিৎসা খরচ কমে B N<br>৩=অন্যান্য (নির্দিষ্ট করুন) C O<br>৪=উপরের কোনটাই না D P	|___| |___| |___| |___|
**অভ্যাস**			
৫০৫	আপনার (নাম) জন্মের পর পরই প্রথম কি খাওয়ানো হয়েছিল?<br><br>**প্রোব করুন: প্রথমেই কি করেছিল - ১টি মাত্র উত্তরে গোল দাগ দিন।**	১=শাল দুধ<br>২=শুধু পানি<br>৩=মিছরী/ চিনি/ গ্লুকোজ পানি<br>৪=মধু<br>৫=সরিষার তেল<br>৬=বুকের দুধ ছাড়া অন্য দুধ<br>৭=অন্য তরল<br>৮=অন্যান্য (নির্দিষ্ট করুন)	|___|
৫০৬	(নাম) কে শালদুধ দেয়া হয়েছিল কি?	১=হ্যাঁ<br>২=না	|___|
৫০৭	আপনার (নাম) জন্মের কতক্ষন পর বুকের দুধ খাওয়া শুরু করেছিল?	মিনিট |___|___| ঘন্টা |___|___|	|___|
		দিন |___|___| ৯৮=কখনোই বুকের দুধ খায়নি<br>৯৯=জানি না	
৫০৮	(নাম) কে বুকের দুধ দেয়ার পূর্বে আপনি কি কোন ধরনের দুধ ফেলে দিয়েছিলেন?	১=হ্যাঁ<br>২=না	|___|
৫০৯	আপনার (নাম) জন্মের সাথে সাথেই কি কেঁদেছিল?	১=হ্যাঁ<br>২=না →	৫১২<br>|___|

		৯=মনে নেই	
৫১০	আপনার (নাম) জন্মের পর কাঁদানো বা শ্বাস নেয়ার জন্য কোন সাহায্যের প্রয়োজন হয়েছিল?	১=হ্যাঁ<br>২=না<br>৯=মনে নেই →	|___| ৫১২
৫১১	কাঁদানোর বা শ্বাস নেয়ার জন্য কি করা হয়েছিল?<br><br>**(উত্তর বলবেন না)**<br><br>**সকল উত্তর রেকর্ড করুন।**	১=কোন উত্তর নাই<br>২=শিশুকে শুকানো হয়েছিল<br>৩=পিঠ ঘষা হয়েছিল<br>৪=পায়ে ঘষা দেয়া হয়েছিল<br>৫=মুখ থেকে মুখে শ্বাস দেয়া হয়েছিল<br>৬=নাড়ি গরম করা হয়েছিল<br>৭=শিশুকে চাপড়ানো হয়েছিল<br>৮=শিশুকে উপর-নীচ করে ধরা হয়েছিল<br>৯=অন্যান্য (নির্দিষ্ট করুন)	|___|
৫১২	নাড়ী কাটার জন্য কি ব্যবহার করা হয়েছিল?	১=সাধারন ব্লেড<br>২=কিটের সাথে দেয়া জীবাণু মুক্ত ব্লেড<br>৩=বাশের চটা/লেইল<br>৪=কাঁচি<br>৫=জীবাণুমুক্ত সার্জিক্যাল ব্লেড<br>৬=স্বাস্থ্যকেন্দ্রে/হাসপাতালে প্রসব<br>৭=অন্যান্য (নির্দিষ্ট করুন) →<br>৯৯=মনে নাই/ জানি না	|___| ৫১৪
৫১৩	যা দ্বারা নাড়ী কাটা হয়েছিল তা কি পানিতে ফুটানো বা জীবানুমুক্ত করা হয়েছিল?	১=হ্যাঁ<br>২=না<br>৯=মনে নেই	|___|
৫১৪	নাড়ী বাধার জন্য কি ব্যবহার করা হয়েছিল?	১=বার্থ কিট থেকে জীবাণু মুক্ত সুতা<br>২=সূতি সুতা<br>৩=নাইলন সুতা<br>৪=জালের সুতা<br>৫=হাসপাতাল/স্বাস্থ্যকেন্দ্রে প্রসব<br>৬=অন্যান্য (নির্দিষ্ট করুন) →<br>৯=মনে নেই	|___| ৫১৬
৫১৫	ব্যবহৃত সূতা কি ফুটানো হয়েছিল ?	১=হ্যাঁ<br>২=না<br>৯=মনে নাই	|___|
৫১৬	নাড়ী কাটা এবং বাধার পর কি কোন কিছু লাগানো হয়েছিল?	১=হ্যাঁ<br>২=না<br>৯=মনে নেই →	|___| ৫১৮
৫১৭	নাড়ী কাটার সাথে সাথে কি লাগানো হয়েছিল ?<br><br>**সকল উত্তর রেকর্ড করুন।**	১=এ্যান্টিবায়োটিক (পাউডার/মলম)<br>২=স্যাভলন<br>৩=ডেটল<br>৪=এলকোহল/স্পিরিট<br>৫=রসুন সহ সরিষার তৈল<br>৬=চাল চিবানো<br>৭=হলুদ	|___|

		৮=আদার রস<br>৯=সিদুঁর<br>১০=বরিক পাউডার<br>১১=জেনশন ভায়োলেট<br>১২=টেলকম পাউডার<br>১৩=অন্যান্য (নির্দিষ্ট করুন)<br>৯৯=জানি না	
৫১৮	জন্মের পর প্রথম (নাম) কে কখন গোসল দেয়া হয়েছিল?	১= জন্মের ২৪ ঘন্টার মধ্যে<br>২=১ম দিন (২৪ ঘন্টা পর)<br>৩=২য় দিন<br>৪=৩য় দিন<br>৫=তারপর<br>৯=মনে নাই	|___|

SECTION 6: NEWBORN CARE: FIRST MONTH & BREAST FEEDING

আমি এখন আপনার (নাম) জন্মের প্রথম মাসে কি ঘটেছিল তা জানার জন্য জিজ্ঞাসা করবো।

৬০১	প্রসবের পর নাড়ী পড়ে যাবার আগে তাতে কি লাগানো হয়েছিল?<br><br>**আর কিছু ?**<br><br>**সকল উত্তর রেকর্ড করুন।**	১=কিছুই না<br>২=এ্যান্টিবায়োটিক (পাউডার/মলম)<br>৩=স্যাভলন<br>৪=ডেটল<br>৫=এলকোহল/স্পিরিট<br>৬=রসুন সহ সরিষার তৈল<br>৭=চাল চিবানো<br>৮=হলুদ<br>৯=আদার রস<br>১০=সিদুঁর<br>১১=বরিক পাউডার<br>১২=জেনশন ভায়োলেট<br>১৩=টেলকম পাউডার<br>১৪=অন্যান্য (নির্দিষ্ট করুন)<br>৯৯=জানি না	|___|
৬০২	আপনার (নাম) জন্মের পর কোন স্বাস্থ্য কর্মী কি তার স্বাস্থ্য চেক-আপ করেছিল?	১=হ্যাঁ<br>২=না<br>৯=মনে নেই →	|___| ৬০৭
৬০৩	আপনার শিশু জন্মের কতদিন বা সপ্তাহ পর প্রথম চেক আপ করিয়েছিলেন?<br><br>**'00' রেকর্ড করুন যদি চেক আপ প্রসবের দিনই করা হয়।**	দিন ঃ প্রসবের পর |___|___| সপ্তাহ ঃ প্রসবের পর |___|___| ৯৯=জানি না/মনে নাই	|___|
৬০৪	কে সেই সময় আপনার (নাম) কে চেকআপ করেছিলেন?	1= Doctors<br>2= Nurse/Midwifes<br>3= Family Welfare Assistant<br>4= Family Welfare Visitor<br>5= Traditional Birth Attendant<br>6= Community Health Care Provider<br>7= Health Assistant	|___|

		8=Village doctor<br>9=Spiritual healer<br>10=Homeopathic<br>11=Kabiraj/Hakim<br>12=NGO worker<br>13= Others (Specify)	
৬০৫	প্রথম চেকআপ কোথায় হয়েছিল ?	1=Home<br>2=H & FWC<br>3=Community Clinic<br>4=UHC South<br>5=UHC North<br>6=Private Clinic Matlab<br>7=MCWC Chandpur<br>8=Private Clinic Chandpur<br>9=Chandpur District Hospital<br>10=Other District Hospital<br>11=Other MCWC<br>12=Other Private Clinic<br>13=Other UHC<br>14=ICMH, Matuail, Dhaka<br>15=NGO<br>16=Others (Specify)	|___|
৬০৬	চেক-আপ করিয়েছিলেন কি বাচ্চার অসুস্থতার জন্য না কি রুটিন চেক-আপ?	১=অসুস্থ<br>২=রুটিন<br>৩=উভয়<br>৯=মনে নাই	|___|

৬০৭	জন্মের ১ মাসের ভিতর আপনার (নামের) নীচের কোন সমস্যা হয়েছিল কি?	জ্বর	১=হ্যাঁ	২=না	৯=জানি না	|___|
		শ্বাস কষ্ট	১=হ্যাঁ	২=না	৯=জানি না	|___|
		জন্ডিস	১=হ্যাঁ	২=না	৯=জানি না	|___|
		ডায়রিয়া	১=হ্যাঁ	২=না	৯=জানি না	|___|
		নাভির সমস্যা	১=হ্যাঁ	২=না	৯=জানি না	|___|
		খিচুনী	১=হ্যাঁ	২=না	৯=জানি না	|___|
		খাওয়ানোর সমস্যা	১=হ্যাঁ	২=না	৯=জানি না	|___|

৬০৮	৬০৭ দেখুনঃ অন্তত: উপরের ১টি সমস্যা	১=হ্যাঁ ২=না	৬১৭<br>|___|
৬০৯	এই সকল সমস্যাগুলোর জন্য আপনি কি কোন সেবা নিয়েছিলেন? বাড়ীতে অথবা অন্য কোথাও ?	১=হ্যাঁ<br>২=না	|___| ৬১৭
৬১০	এ সমস্যাগুলোর জন্য প্রথম কোথায় সেবা নিয়েছিলেন?<br><br>**(জন্মের প্রথম মাসের সমস্যার জন্য)**<br><br>একটি মাত্র উত্তর রেকর্ড করুন যেখানে মহিলা / তার পরিবার **প্রথম** সেবা নিয়েছিল।	1=Home<br>2=H & FWC<br>3=Community Clinic<br>4=UHC South<br>5=UHC North<br>6=Private Clinic Matlab<br>7=MCWC Chandpur	|___|

		8=Private Clinic Chandpur<br>9=Chandpur District Hospital<br>10=Other District Hospital<br>11=Other MCWC<br>12=Other Private Clinic<br>13=Other UHC<br>14=ICMH, Matuail, Dhaka<br>15=NGO<br>16=Matlab icddr,b<br>17=Pharmacy<br>18=Homeopathic<br>19=Others (Specify)	
৬১১	কে এই সেবা / চিকিৎসা দিয়েছিলেন ?<br><br>**(জন্মের প্রথম মাসের সমস্যার জন্য)**<br><br>একটি মাত্র উত্তর রেকর্ড করুন যার কাছ থেকে মহিলা / তার পরিবার **প্রথম** সেবা পেয়েছিল।	1= Doctors<br>2= Nurse/Midwifes<br>3= Family Welfare Assistant<br>4= Family Welfare Visitor<br>5= Traditional Birth Attendant<br>6= Community Health Care Provider<br>7= Health Assistant<br>8=Village doctor/ Pharmacy<br>9=Spiritual healer<br>10=Homeopathic<br>11=Kabiraj/Hakim<br>12=NGO worker<br>13= Others (Specify)	|___|
৬১২	আপনার (নাম) কি ধরনের চিকিৎসা পেয়েছিল ?<br><br>**(জন্মের প্রথম মাসের সমস্যার জন্য)**<br><br>**সকল উত্তর রেকর্ড করুন।**	১=ফার্মেসী থেকে ঔষধ<br>২=FWV/CHCP থেকে রেফারেল<br>৩=FWV/CHCP থেকে পরামর্শ<br>৪=পানি পড়া/দ্রব্য<br>৫=তাবিজ<br>৬=হোমিও ঔষধ<br>৭=শিরায় স্যালাইন<br>৮=ডাক্তার/নার্স এর নিকট হতে ঔষধ<br>৯=কবিরাজি/হেকিমি ঔষধ<br>১০=অন্যান্য (নির্দিষ্ট করুন) ____________________	|___|

৬১৩	এই সমস্যার চিকিৎসার জন্য আপনার বাচ্চাকে কি কোথাও রেফার করা হয়েছিল ?<br>**(জন্মের প্রথম মাসের সমস্যার জন্য)**	১=হ্যাঁ<br><br>২=না	|___| <br>৬১৭
৬১৪	কোথায় আপনার বাচ্চাকে রেফার করা হয়েছিল?<br><br>**(জন্মের প্রথম মাসের সমস্যার জন্য)**	1=Home<br>2=H & FWC<br>3=Community Clinic<br>4=UHC South	|___|

		5=UHC North<br>6=Private Clinic Matlab<br>7=MCWC Chandpur<br>8=Private Clinic Chandpur<br>9=Chandpur District Hospital<br>10=Other District Hospital<br>11=Other MCWC<br>12=Other Private Clinic<br>13=Other UHC<br>14=ICMH, Matuail, Dhaka<br>15=NGO<br>16=Matlab icddr,b<br>17=Others (Specify)	
৬১৫	আপনি কি রেফার্ড স্থানে গিয়েছিলেন ?<br>**(জন্মের প্রথম মাসের সমস্যার জন্য)**	১=হ্যাঁ<br>২=না	৬১৭<br>|__|
৬১৬	যদি রেফারকরা স্থানে না যেয়ে থাকেন তবে তার কারণ কি?<br>**(জন্মের প্রথম মাসের সমস্যার জন্য)**<br>**সকল উত্তর রেকর্ড করুন।**	১=মা প্রয়োজন মনে করেন নি →<br>২=স্বামী/পরিবার প্রয়োজন মনে করেন নি<br>৩= অন্য প্রয়োজন ছিল<br>৪=সেবা কেন্দ্র অনেক দুরে<br>৫=কোন যানবাহন ছিল না<br>৬=শিশুদের দেখার কেউ ছিল না<br>৭=খরচ অনেক বেশী<br>৮=সেবা মানসম্মত নয়<br>৯=জানতাম না কোথায় যেতে হবে<br>১০=যাবার সময় ছিল না<br>১১=টাকা যোগার করা সহজ নয়<br>১২=অন্যান্য (নির্দিষ্ট করুন)<br>৯৯=জানি না	|__|
**এখন আমি আপনাকে বাচ্চার খাবারের ধরণ সম্পর্কে প্রশ্ন করবো:**			
৬১৭	কত মাস পর্যন্ত আপনি বাচ্চাকে বুকের দুধ দিয়েছিলেন ?	মাস |__|__| দিন |__|__| ৯৬= কখনও বুকের দুধ দেয় নাই<br>৯৮= এখনও বুকের দুধ খায়<br>৯৯= জানি না	|__|
৬১৮	বুকের দুধ ছাড়া আপনার বাচ্চাকে কি কখনোই কোন শক্ত বা নরম (Semi-Solid) কিছু খেতে / পান করতে দিয়েছেন?	১=হ্যাঁ<br>২=না	|__|

৬১৯	কোন বয়সে বাচ্চাকে বুকের দুধ ছাড়া প্রথম অন্য খাবার/পানীয় দিয়েছিলেন?	মাস |__|__| দিন |__|__| ৯৯=জানি না	|__|
৬২০	বুকের দুধ ছাড়া আপনার বাচ্চাকে আর কি দিয়েছেন?<br>**সকল উত্তর রেকর্ড করুন।**	১=শুধু পানি<br>২=চিনির সরবৎ/মধু/ফলের রস<br>৩=বাচ্চার গুঁড়ো দুধ<br>৪=গরু/ছাগলের দুধ	|__|

		৫=অন্য তরল খাদ্য<br>৬=কলা/পেপে/আম<br>৭=সবুজ শাকসবজী<br>৮=ভাত, গম, সুজি<br>৯=মাংস/মাছ/ডিম<br>১০=ডাল<br>১১=চা<br>১২=এলোপেথিক ঔষধ<br>১৩=ওরস্যালাইন<br>১৪=খিচুড়ী<br>১৫=অন্যান্য (নির্দিষ্ট করুন)<br>১৬=কিছুই না	
৬২১	আপনার বাচ্চাকে বুকের দুধ ছাড়া অন্য খাবার / পানীয় কেন দিয়েছিলেন?<br><br>**সকল উত্তর রেকর্ড করুন।**	১=বাচ্চার বয়স ৬ মাসের বেশী<br>২=বুকে পর্যাপ্ত দুধ ছিল না<br>৩=বুকের দুধ বাতাস লেগে নষ্ট হয়ে গেছে<br>৪=দুধ খাওয়ালে পাতলা পায়খানা হতো<br>৫=দাই/ডাক্তার নিষেধ করেছে<br>৬=বুকের দুধ খুবই পাতলা<br>৭=বাচ্চা বড় হয়েছে অতিরিক্ত খাবার দরকার<br>৮=শুধু মাত্র বুকের দুধ খাওয়ানোর উপকারিতা জানে না<br>৯=অন্যান্য (নির্দিষ্ট করুন)	|__|
৬২২	দেখুন ১০৩ঃ বাচ্চা জীবিত	১=হ্যাঁ ২=না	৭০১<br>|__|
৬২৩	গত ২৪ ঘন্টায় আপনার বাচ্চা কতবার বুকের দুধ ছাড়া অন্য শক্ত বা নরম (Semi-Solid or Soft) খাবার খেয়েছে?<br>**যদি 7 বার বা তার বেশী হয় রেকর্ড করুন 7**	|__|__| [illegible]র	|__|

৬২৪ গত ২৪ ঘন্টায় বাচ্চাকে নীচের খাবারের কোন একটি কি দিয়েছিলেন?	পানি	১=হ্যাঁ	২=না	|__|
	চিনির সরবৎ / মধু/ফলের র[illegible]	১=হ্যাঁ	২=না	|__|
	বাচ্চার গুঁড়ো দুধ	১=হ্যাঁ	২=না	|__|
	গরু / ছাগলের দুধ	১=হ্যাঁ	২=না	|__|
	অন্য তরল খাদ্য	১=হ্যাঁ	২=না	|__|
	কলা / পেপে / আম	১=হ্যাঁ	২=না	|__|
	সবুজ শাক-সবজী	১=হ্যাঁ	২=না	|__|
	ভাত, গম, সুজি	১=হ্যাঁ	২=না	|__|
	মাংস / মাছ / ডিম	১=হ্যাঁ	২=না	|__|
	ডাল	১=হ্যাঁ	২=না	|__|
	চা	১=হ্যাঁ	২=না	|__|
	এলোপ্যাথিক ঔষধ	১=হ্যাঁ	২=না	|__|
	ওরস্যালাইন	১=হ্যাঁ	২=না	|__|
	খিচুড়ী	১=হ্যাঁ	২=না	|__|
	অন্যান্য (নির্দিষ্ট করুন)	১=হ্যাঁ	২=না	|__|

SECTION 7: PERCEPTIONS OF LOCAL FACILITY (স্থানীয় সুবিধা সম্পর্কে উপলব্ধি)

৭০১	আপনি কি আপনার এলাকার কোন স্বাস্থ্যকেন্দ্র / হাসপাতালের নাম জানেন যেখানে একজন মহিলা স্বাস্থ্য সমস্যা বা সন্তান প্রসবের জন্য [illegible]	১=হ্যাঁ<br>২=না	|__| ৮০১

	যেতে পারেন ?		
৭০২	যদি জানেন: সেটা কোথায় ?<br><br>**সকল উত্তর রেকর্ড করুন।**	1=H & FWC →<br>2=Community Clinic<br>3=UHC South<br>4=UHC North<br>5=Private Clinic Matlab<br>6=MCWC Chandpur<br>7=Private Clinic Chandpur<br>8=Chandpur District Hospital<br>9=Other District Hospital<br>10=Other MCWC<br>11=Other Private Clinic<br>12=Other UHC<br>13=ICMH, Matuail, Dhaka<br>14=NGO<br>15=Others (Specify)	|__|
৭০৩	যদি একটির বেশী সেবা কেন্দ্রের নাম বলে থাকেন তাহলে সবচেয়ে কাছে কোনটি?	1=H & FWC<br>2=Community Clinic<br>3=UHC South<br>4=UHC North<br>5=Private Clinic Matlab<br>6=MCWC Chandpur<br>7=Private Clinic Chandpur<br>8=Chandpur District Hospital<br>9=Other District Hospital<br>10=Other MCWC<br>11=Other Private Clinic<br>12=Other UHC<br>13=ICMH, Matuail, Dhaka<br>14=NGO<br>15=Others (Specify)	|__|
৭০৪	যদি হাসপাতাল / স্বাস্থ্যকেন্দ্রের সেবাদানকারীর কোন সেবা আপনার ভাল লেগে থাকে তাহলে তা কি কি?<br><br>**প্রোব করুন : আর কিছু ?**<br><br>**সকল উত্তর রেকর্ড করুন।**	১=ভাল ব্যবহার<br>২=ঔষধ সরবরাহ পাওয়া<br>৩=অপেক্ষা করতে হয়নি সেবার জন্য<br>৪=চিকিৎসা কার্যকরী ছিল<br>৫=আপনার প্রশ্নের উত্তর দেওয়া<br>৬=খরচ আওতার মধ্যে	|__|
		৭=পরিস্কার<br>৮=বন্ধুভাবাপন্ন এবং কথা বার্তায় ভাল<br>৯=অন্যান্য (নির্দিষ্ট করুন) ________________<br>১০=জানি না/যাই নাই	
৭০৫	যদি হাসপাতাল / স্বাস্থ্য কেন্দ্রের সেবাদানকারীর কোন সেবা আপনার ভাল না লেগে থাকে তাহলে তা কি কি?	১=খারাপ ব্যবহার<br>২=ঔষধ না পাওয়া<br>৩=দেরিতে চিকিৎসা শুরু করা	|__|

	প্রোব করুন: আরও কিছু।<br><br>সকল উত্তর রেকর্ড করুন।	৪=খরচ অনেক বেশী/সামর্থের বাইরে<br>৫=জিজ্ঞাসা করলে উত্তর না দেয়া<br>৬=অপরিস্কার<br>৭=বন্ধুভাবাপন্ন না<br>৮=চিকিৎসায় ভাল হয় নি<br>৯=অন্যান্য (নির্দিষ্ট করুন) ____________<br>১০=জানি না/যাই নাই	
৭০৬	আপনি এই স্বাস্থ্যকেন্দ্রে বা হাসপাতালের সেবা সম্পর্কে কিভাবে জানেন?<br><br>সকল উত্তর রেকর্ড করুন।	১=আগেও এইস্থানে স্বাস্থ্য সেবা নিয়েছেন<br>২=পরিবারের কাছ থেকে জেনেছেন<br>৩=বন্ধুদের কাছ থেকে জেনেছেন<br>৪=অন্যান্য (নির্দিষ্ট করুন) ____________<br>৯৯=জানেনা	|__|

SECTION 8: SOCIO-ECONOMIC STATUS (SES)

CID of Household Head	|__|__|__|__|__|__|__|__|__|
RID of Household Head	|__|__|__|__|__|__|__|__|__|__|
CID of respondent if s/he is not Household Head	|__|__|__|__|__|__|__|__|__|

জমি-জমা সংক্রান্ত তথ্য:

		শতক	কানি	গন্ডা	কড়া
৮০১	আপনার খানায় জমির পরিমান কত?				
	বসত জমি (পুকুর/ডোবা সহ)	|__|__|__|__|	|__|__|__|__|	|__|__|__|__|	|__|__|__|__|
	কৃষি জমি (নিজের)	|__|__|__|__|	|__|__|__|__|	|__|__|__|__|	|__|__|__|__|

১ কানি= শতক, ১ গন্ডা= শতক, ১ কড়া= শতক

আয়ের উৎস:

৮০২	(a) গত ১ বছরে (১২ মাস) আপনার খানায় আয়ের উৎস কি কি ছিল?	১	কৃষি (নিজের জমি)	১=হ্যাঁ	২=না	|__|
		২	কৃষি (বর্গা ফসল)	১=হ্যাঁ	২=না	|__|
		৩	জমি/পুকুর গচ্ছিত/চুক্তিকৃত/ভাড়া দেয়া/নেয়া)	১=হ্যাঁ	২=না	|__|
	**সকল উত্তর রেকর্ড করুন।**	৪	দিনমজুর/রিক্সা চালক/ভ্যান চালক	১=হ্যাঁ	২=না	|__|
		৫	দক্ষ শ্রমিক (ড্রাইভার/কাঠমিস্ত্রি/সেনিটারী মিস্ত্রি/ইলেকট্রিক মিস্ত্রি)	১=হ্যাঁ	২=না	|__|
		৬	মাছ ধরে বিক্রি করে/মাছ চাষ	১=হ্যাঁ	২=না	|__|
		৭	গবাদি পশু/হাস-মুরগী/পোল্ট্রি খামার/দুধ বিক্রি	১=হ্যাঁ	২=না	|__|
		৮	কুটির শিল্প/হস্ত শিল্প	১=হ্যাঁ	২=না	|__|
		৯	দর্জি	১=হ্যাঁ	২=না	|__|
		১০	ক্ষুদ্র ব্যবসা	১=হ্যাঁ	২=না	|__|
		১১	মাঝারী ব্যবসা	১=হ্যাঁ	২=না	|__|
		১২	ব্যবসা (বিভিন্ন ধরনের বড় ব্যবসা)	১=হ্যাঁ	২=না	|__|
		১৩	চাকুরী	১=হ্যাঁ	২=না	|__|
		১৪	পেনশন	১=হ্যাঁ	২=না	|__|
	(b) আয়ের প্রধান উৎস কি ছিল?	১৫	দেশের মধ্যে থেকে টাকা	১=হ্যাঁ	২=না	|__|
	Sl No. |__|__|	১৬	বিদেশ থেকে টাকা	১=হ্যাঁ	২=না	|__|
		১৭	কাজের বিনিময়ে খাদ্য	১=হ্যাঁ	২=না	|__|
		১৮	বয়স্ক ভাতা/দুস্থ ভাতা/ভিজিডি ভাতা/মুক্তিযোদ্ধা ভাতা/চেয়ারম্যান/মেম্বার	১=হ্যাঁ	২=না	|__|
		১৯	বাড়ি/দোকান ভাড়া	১=হ্যাঁ	২=না	|__|

		২০	ট্যাক্সি/টেম্পো/অটো ভাড়া থেকে আয়	১=হ্যাঁ	২=না	|__|
		২১	নৌকা/রিক্সা/ভ্যান ভাড়া থেকে আয়	১=হ্যাঁ	২=না	|__|
		২২	সুদের ব্যবসা	১=হ্যাঁ	২=না	|__|
		২৩	সাংবাদিক	১=হ্যাঁ	২=না	|__|
		২৪	অন্যান্য (উল্লেখ করুন)			

খাদ্য অনিশ্চয়তা:

৮০৩	গত ১ বছরে আপনার খানায় সকলের তিনবেলা খাবারের ব্যবস্থা ছিল কি-না?	১=হ্যাঁ	২=না	|__|

বাসস্থান:

৮০৪	(a) আপনার খানায় কয়টি বাসঘর আছে? (সংখ্যায় উল্লেখ করুন)					|__|__|
	(b) প্রধান বসতঘর কি দিয়ে তৈরী?	**উপকরণ**	**ছাদ**	**দেয়াল**	**মেঝে**	
		পাকা	১	১	১	|__|
		টিন	২	২	২	|__|
		টিন ও বাঁশ	৩	৩	৩	|__|
	**(নিজে দেখে লিখুন)**	টিন ও কাঠ	৪	৪	৪	|__|
		টিন ও অন্যান্য	৫	৫	৫	|__|
		বাঁশ ও অন্যান্য	৬	৬	৬	|__|
		কাঁচা (মাটি)	৭	৭	৭	|__|
		কাঠ	৮	৮	৮	|__|
		অন্যান্য	৯	৯	৯	|__|

ব্যবহৃত জিনিসপত্র/সম্পদ:

৮০৫	আপনার বাসার কেউ কি নীচের জিনিসগুলি ব্যবহার করেন?	মাছ ধরার জাল	১=হ্যাঁ	২=না	|__|
		মাছ ধরার নৌকা	১=হ্যাঁ	২=না	|__|
		নৌকা	১=হ্যাঁ	২=না	|__|
	**সকল উত্তর রেকর্ড করুন।**	দোকান (মালিকানা)	১=হ্যাঁ	২=না	|__|
		রিক্সা/ভ্যান	১=হ্যাঁ	২=না	|__|
		অটো রিক্সা/ইঞ্জিন চালিত রিক্সা/সিএনজি	১=হ্যাঁ	২=না	|__|
		পাওয়ার ট্রিলার (চাষাবাদে ব্যবহৃত)	১=হ্যাঁ	২=না	|__|
		পাম্প (সেচ কাজ/পানি উত্তোলনে ব্যবহৃত)	১=হ্যাঁ	২=না	|__|
		ধান মাড়ানোর মেশিন	১=হ্যাঁ	২=না	|__|
		চাষাবাদে ব্যবহৃত যন্ত্রপাতি	১=হ্যাঁ	২=না	|__|
		আইপিএস	১=হ্যাঁ	২=না	|__|
		কম্পিউটার	১=হ্যাঁ	২=না	|__|
		জেনারেটর	১=হ্যাঁ	২=না	|__|
		সৌর বিদুৎ	১=হ্যাঁ	২=না	|__|
		টিউবওয়েল (অগভীর নলকূপ)	১=হ্যাঁ	২=না	|__|
		টিউবওয়েল (গভীর নলকূপ)	১=হ্যাঁ	২=না	|__|
		কম্বল	১=হ্যাঁ	২=না	|__|
		লেপ-তোষক	১=হ্যাঁ	২=না	|__|
		চেয়ার-টেবিল	১=হ্যাঁ	২=না	|__|
		ডাইনিং টেবিল	১=হ্যাঁ	২=না	|__|
		আলমিরা/শোকেস	১=হ্যাঁ	২=না	|__|
		সোফা সেট	১=হ্যাঁ	২=না	|__|
		টেলিভিশন	১=হ্যাঁ	২=না	|__|
		রেডিও/সিডি/টেপ রেকর্ডার/ডিভিডি	১=হ্যাঁ	২=না	|__|

		ল্যান্ডফোন/মোবাইল ফোন	১=হ্যাঁ	২=না	|__|
		বাই সাইকেল	১=হ্যাঁ	২=না	|__|
		মোটর সাইকেল	১=হ্যাঁ	২=না	|__|
		ফ্রিজ	১=হ্যাঁ	২=না	|__|
		ফ্যান	১=হ্যাঁ	২=না	|__|
		সেলাই মেশিন	১=হ্যাঁ	২=না	|__|
		অন্যান্য (উল্লেখ করুন)			

গবাদি পশুপালন:

৮০৬	আপনার খানায় কোন গবাদি পশু আছে? (গরু-ছাগল, হাস-মুরগী)		১=হ্যাঁ	২=না	|__|
৮০৭	যদি হ্যাঁ হয়, তাহলে প্রকার এবং সংখ্যায় উল্লেখ করুন?		প্রকার		সংখ্যা
		১	হাঁস-মুরগী		|__|__|
		২	ছাগল-ভেড়া		|__|__|
		৩	গরু-মহিষ		|__|__|

পয়: নিস্কাশন ব্যবস্থা:

৮০৮	আপনার নিজের কোন পায়খানা আছে কি?		১=হ্যাঁ	২=না	|__|
৮০৯	আপনারা কি ধরনের পায়খানা ব্যবহার করেন?	স্যানেটারী ল্যাট্রিন	১=হ্যাঁ	২=না	|__|
		রিং/স্লাব কিন্তু পায়খানা বাহিরে যায় না	১=হ্যাঁ	২=না	|__|
		রিং/স্লাব কিন্তু পায়খানা বাহিরে যায়	১=হ্যাঁ	২=না	|__|
	**(নিজে দেখে লিখুন)**	পাকা পায়খানা কিন্তু পায়খানা বাহিরে যায়	১=হ্যাঁ	২=না	|__|
		কাঁচা পায়খানা	১=হ্যাঁ	২=না	|__|
		খোলা মাঠ/ঝোঁপ-ঝাড়	১=হ্যাঁ	২=না	|__|
		অন্যান্য (উল্লেখ করুন)			

পানীয় জলের উৎস: (১ বছরের মধ্যে)

৮১০	আপনার খানায় ফিল্টার/বিশুদ্ধ পানির ব্যবস্থা আছে কি?		১=হ্যাঁ	২=না	|__|
৮১১	আপনাদের খাবার পানির প্রধান উৎস কি?	ডিপ টিউবওয়েল (গভীর নলকূপ)	১=হ্যাঁ	২=না	|__|
		টিউবওয়েল (সবুজ)	১=হ্যাঁ	২=না	|__|
		টিউবওয়েল (লাল)	১=হ্যাঁ	২=না	|__|
		টিউবওয়েল (এখনো পরীক্ষা করা হয়নি)	১=হ্যাঁ	২=না	|__|
	**(বেশীর ভাগ ক্ষেত্রে ব্যবহৃত)**	সাপ্লাই পানি	১=হ্যাঁ	২=না	|__|
		বৃষ্টির পানি	১=হ্যাঁ	২=না	|__|
		পুকুর	১=হ্যাঁ	২=না	|__|
		নদী	১=হ্যাঁ	২=না	|__|
		ডোবা/খাল	১=হ্যাঁ	২=না	|__|
		অন্যান্য (উল্লেখ করুন)			

আলোর উৎস:

৮১২	আপনার বাড়িতে আলোর প্রধান উৎস কি?	কেরোসিন	১=হ্যাঁ	২=না	|__|
		বিদ্যুৎ	১=হ্যাঁ	২=না	|__|
	**সকল উত্তর রেকর্ড করুন।**	সৌর বিদ্যুৎ	১=হ্যাঁ	২=না	|__|
		জেনারেটর	১=হ্যাঁ	২=না	|__|
		অন্যান্য (উল্লেখ করুন)			

জ্বালানী দ্রব্য:

৮১৩	আপনারা রান্নার জন্য প্রধানত: কি ব্যবহার করেন?	কাঠ/কাঠের গুড়া/ধানের তুস	১=হ্যাঁ	২=না	|___|
		পাতা ও খড়	১=হ্যাঁ	২=না	|___|
		কেরোসিন (স্টোভ)	১=হ্যাঁ	২=না	|___|
	**সকল উত্তর রেকর্ড করুন।**	গ্যাস (লাইন)	১=হ্যাঁ	২=না	|___|
		গ্যাস (সিলিন্ডার)	১=হ্যাঁ	২=না	|___|
		বিদ্যুৎ (ওভেন/রাইস কুকার/মাইক্রোওভেন)	১=হ্যাঁ	২=না	|___|
**এন.জি.ও/সমিতি:**					
৮১৪	আপনার খানায় কেউ কোন সমিতি বা এন.জি.ও-র সদস্য কি-না?		১=হ্যাঁ	২=না	|___|
৮১৫	আপনার খানায় কেউ ঋণ নিয়েছেন কি-না? (৬ মাসের মধ্যে)		১=হ্যাঁ	২=না	|___|
৮১৬	যদি হ্যাঁ হয়, তাহলে নিয়মিত ঋণ পরিশোধ করতে পারছেন কি-না?		১=হ্যাঁ	২=না	|___|
**ব্যাংক একাউন্ট:**					
৮১৭	আপনি কিংবা খানার অন্য কারো ব্যাংক একাউন্ট আছে কি-না?		১=হ্যাঁ	২=না	|___|

End time: |__|__|:|__|__|

Eddited By: **Code:** |___|___|

Date: |___|___| |___|___| |___|___|

Entry By: **Code:** |___|___|

Date: |___|___| |___|___| |___|___|
