# Supplementary material for: Level and determinants of birth preparedness and complication readiness among pregnant women: A cross sectional study in a rural area in Bangladesh
Source: PLoS One. 2018 Dec 17;13(12):e0209076. doi: 10.1371/journal.pone.0209076 (PMC6296737; doi:10.1371/journal.pone.0209076)
Supplement: S1 Table — (PDF) [file pone.0209076.s003.pdf]

Variables	Recall Period	
	≤12 months	>12 months
	Adjusted OR (95%CI)	Adjusted OR (95%CI)
Asset index		
One (Poorest)	1.0	1.0
Two	1.89(.91-3.95)	1.80(.83-3.88)
Three	2.34(1.13-5.10)	2.08(.930-4.66)
Four	3.70(1.78-7.70)	1.88(.769-4.60)
Five (Richest)	4.55(2.27-9.12)	3.58(1.68-7.64)
No. of antenatal care visits		
0-1	1.0	1.0
2-3	2.59(1.40-4.81)	2.32 (1.20-4.47)
≥ 4	5.94 (3.25-10.85)	6.04(3.16-11.53)
Knowledge of danger signs during pregnancy		
Poor Knowledge	1.0	1.0
Good Knowledge	1.5 (.99-2.26)	2.6 (1.64-4.12)
Knowledge of danger signs during delivery		
Poor Knowledge	1.0	1.0
Good Knowledge	1.95(1.30-2.92)	1.46(.92-2.33)
